# Copper‐loaded Milk‐Protein Derived Microgel Preserves Cardiac Metabolic Homeostasis After Myocardial Infarction

**DOI:** 10.1002/advs.202401527

**Published:** 2024-07-15

**Authors:** Xiaoqian Hong, Geer Tian, Binyao Dai, Xuhao Zhou, Ying Gao, Lianlian Zhu, Haoran Liu, Qinchao Zhu, Liwen Zhang, Yang Zhu, Daxi Ren, Chengchen Guo, Jinliang Nan, Xianbao Liu, Jian'an Wang, Tanchen Ren

**Affiliations:** ^1^ Department of Cardiology The Second Affiliated Hospital School of Medicine Zhejiang University Hangzhou 310009 China; ^2^ State Key Laboratory of Transvascular Implantation Devices Heart Regeneration and Repair Key Laboratory Zhejiang Province Hangzhou 310009 China; ^3^ Binjiang Institute of Zhejiang University Hangzhou 310053 China; ^4^ MOE Key Laboratory of Macromolecular Synthesis and Functionalization Department of Polymer Science and Engineering Zhejiang University Hangzhou 310027 China; ^5^ School of Engineering Westlake University Hangzhou 310023 China; ^6^ Institute of Dairy Science College of Animal Sciences Zhejiang University Hangzhou 310027 China

**Keywords:** casein, copper, hydrogels, metabolic homeostasis, myocardial infarctions

## Abstract

Myocardial Infarction (MI) is a leading cause of death worldwide. Metabolic modulation is a promising therapeutic approach to prevent adverse remodeling after MI. However, whether material‐derived cues can treat MI through metabolic regulation is mainly unexplored. Herein, a Cu^2+^ loaded casein microgel (CuCMG) aiming to rescue the pathological intramyocardial metabolism for MI amelioration is developed. Cu^2+^ is an important ion factor involved in metabolic pathways, and intracardiac copper drain is observed after MI. It is thus speculated that intramyocardial supplementation of Cu^2+^ can rescue myocardial metabolism. Casein, a milk‐derived protein, is screened out as Cu^2+^ carrier through molecular‐docking based on Cu^2+^ loading capacity and accessibility. CuCMGs notably attenuate MI‐induced cardiac dysfunction and maladaptive remodeling, accompanied by increased angiogenesis. The results from unbiased transcriptome profiling and oxidative phosphorylation analyses support the hypothesis that CuCMG prominently rescued the metabolic homeostasis of myocardium after MI. These findings enhance the understanding of the design and application of metabolic‐modulating biomaterials for ischemic cardiomyopathy therapy.

## Graphical abstract

Ischemic myocardium is featured with metabolic disequilibrium and copper depletion. This study raised a material‐based therapeutic strategy by intramyocardial supplement of Cu^2+^ to rescue myocardial metabolism. The milk‐derived casein microgel is screened out as Cu^2+^ carrier through molecular‐docking by loading capacity and accessibility. The Cu^2+^ loaded microgel prominently rescued cardiac functions by preserving metabolic homeostasis.

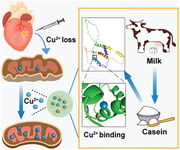

## 1 Introduction

Myocardial infarction (MI) is one of the major causes of morbidity and mortality worldwide.^[^ ^1^, ^2^ ^]^ Despite significant progress in treatment and technology to revascularize the coronary artery that has been developed in recent years, MI still poses a high global medical burden due to the following ischemic cardiomyopathy.^[^ ^3^, ^4^ ^]^ Heart function is sensitive to energy supply.^[^ ^5^ ^]^ MI leads to a sudden cessation of local blood and oxygen supply which changes the level of oxidative phosphorylation (OXPHOS). The disorder of myocardial energy metabolism after ischemia ultimately leads to changes in left ventricular function and structure, increasing the probability of cardiac remodeling and heart failure.^[^ ^6^ ^]^

Strategies that regulate metabolic homeostasis after MI are promising therapeutic candidates against heart failure. Although no medication has been applied clinically to modulate cardiac metabolism directly in clinically acute MI, more and more results have been presented to rescue heart failure by reversing the detrimental metabolic processes in ischemic cardiomyopathy. Inhibitors and activators of certain metabolic pathways have shown cardioprotection effects in various studies.^[^ ^7^, ^8^, ^9^ ^]^ However, drug toxicity remains a challenge due to the risks of altering systemic metabolic stability.^[^ ^10^ ^]^ More recently, certain long non‐coding RNA^[^ ^11^ ^]^ and circular RNA^[^ ^12^ ^]^ have been identified to attenuate mitochondria malfunction after MI. Adeno‐associated virus (AAV), which carried certain genes to edit metabolic pathways in cardiomyocytes,^[^ ^13^ ^]^ achieved protective effects on cardiac functions in mice with left anterior descending (LAD) branch ligation.^[^ ^14^ ^]^ However, AAVs have to be administered weeks before the ischemic events to allow sufficient transfection, which may be less clinically relevant as patients cannot be treated beforehand.^[^ ^15^ ^]^

Bioengineering strategies based on materials have the potential to overcome the systemic side effects and take effect immediately upon administration for metabolic regulation.^[^ ^16^, ^17^, ^18^ ^]^ The delivery of inherent metabolic factors based on bioengineering cargoes can enhance tissue regeneration with high performance.^[^ ^19^ ^]^ Gentaro Ikeda et al. directly transferred mitochondria to infarct heart tissue by injecting mitochondria‐rich extracellular vesicles (EVs), successfully restored myocardial bioenergy, and enhanced cardiac function after MI.^[^ ^20^ ^]^ Pengfei Chen et al. developed a plant‐derived photosynthetic system based on nanothylakoid units encapsulated in the cell membrane to improve anabolism in degenerated chondrocytes.^[^ ^21^ ^]^ More user‐friendly materials, such as scaffolds that can continuously release regulatory metabolites, i.e., citrate^[^ ^22^ ^]^ and succinate,^[^ ^23^ ^]^ have been developed to modulate energy metabolism for bone regeneration. Thus, we speculate that materials loading with metabolic modulation factors may exert positive results to enhance cardiac metabolic homeostasis and reconstruction.

Copper (Cu) is an essential trace element that plays an important role in metabolic processes.^[^ ^24^, ^25^, ^26^ ^]^ They are cofactors of many key mitochondrial enzymes (i.e., cytochrome c oxidase (CCO) and superoxide dismutase 1 (SOD1)), all of which play important roles in maintaining mitochondrial metabolic homeostasis.^[^ ^27^ ^]^ Cu depletion has been reported to affect mitochondrial OXPHOS and impair metastasis in tumors.^[^ ^28^ ^]^ In the case of MI, Cu level in the ischemic myocardium decreased significantly after LAD branch ligation in mice, which did not recover over time within 28 days.^[^ ^29^ ^]^ Therapeutic methods emphasized in Cu supplementation have shown promising results. In these researches, the functions of Cu involved in several mechanisms, such as suppressing the transformation of fibroblasts into myofibroblasts,^[^ ^30^ ^]^ increasing the stability of hypoxia‐inducible factor‐1 (HIF‐1),^[^ ^31^ ^]^ acting as a catalyst to enhance the local release of nitride oxide which increase endothelial cell activity.^[^ ^32^ ^]^ However, it is unclear whether supplementing Cu^2+^ can rescue myocardial metabolism after infarction.

As the serum level of Cu elevated after MI,^[^ ^33^ ^]^ oral Cu supplementation or intravascular injection may induce potential side effects, i.e., systemic toxicity. Besides, it is hard to control the dosage of Cu through dietary uptakes. The Cu overload caused by dietary supplementation induced endoplasmic reticulum stress and mitochondrial damage, which even led to myocardial cell apoptosis.^[^ ^34^ ^]^ Local administration is an effective method to increase therapeutic functions and decrease systemic side effects. However, the difficulty of heart delivery requests a highly controllable release of Cu in order to rescue the long‐term Cu deficiency with a single dose.

Natural proteins have many advantages as drug carriers, such as good biocompatibility, abundant sources, safety, clear components and structures for interaction prediction, and usually strong interactions with metal ions. Protein‐based biomaterials have multiple reaction sites and can be made into various forms for in vivo tissue engineering.^[^ ^35^, ^36^ ^]^ Among them, microgels have received widespread attention as carriers for therapeutic factors due to the feasibility of minimally invasive delivery and stable retention for controllable release.^[^ ^37^, ^38^ ^]^ The size, shape, surface properties, and composition of the microgel can be controlled by the preparation process, according to the requirements of various applications.^[^ ^39^, ^40^, ^41^ ^]^ Crosslinkers such as glutaraldehyde and genipin are usually used to prepare protein microgels without the demand for molecular premodification,^[^ ^42^ ^]^ however, the residual cross–linkers are usually cytotoxic, which may lead to adverse effects after implantation. Methacrylate‐modified proteins are also widely used for microgel fabrication because of the high controllability,^[^ ^43^ ^]^ however, the side chain modification may alter protein structure, and lead to an increase in preparing time and effort. Previously, we used a ruthenium/sodium persulfate (Ru/SPS) photocross–linking system to form a hydrogel by forming a bi‐tyrosine bond between tyrosine residues in protein molecules, without the necessity for pre‐modification.^[^ ^44^ ^]^ We thus speculate Ru/SPS system can also be used for microgel formation.

**Figure 1:**
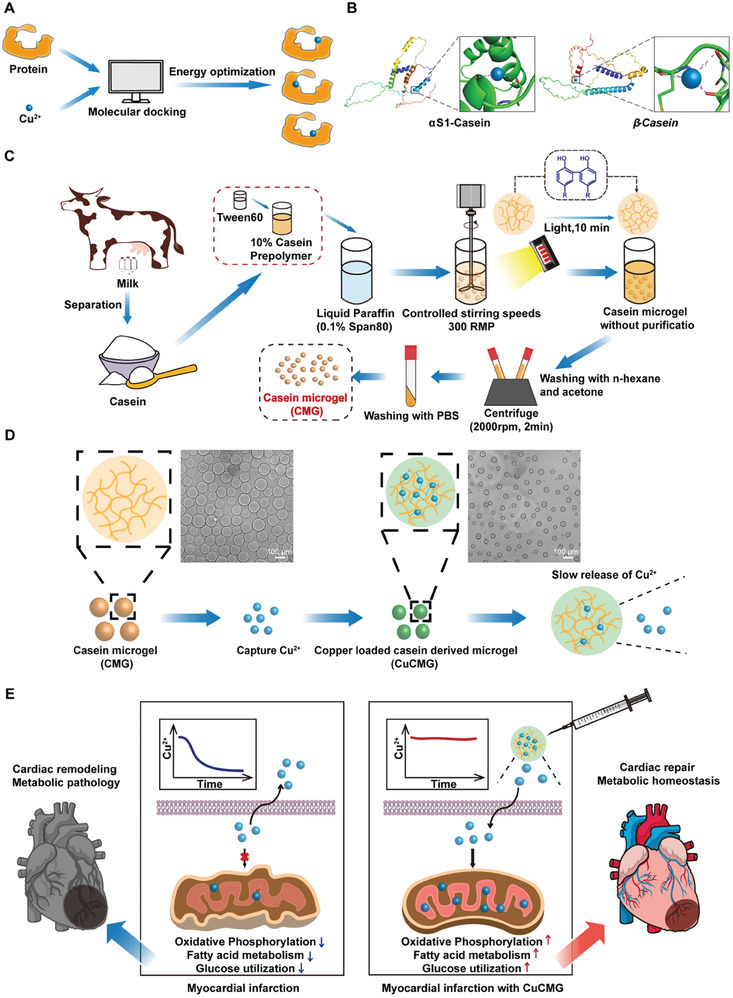
Schematic illustration of Cu‐loaded milk‐protein derived microgel with protective effects on cardiac metabolic homeostasis to improve myocardial infarction. A) Schematic diagram of molecular docking simulation. B) Schematic diagram of molecular docking between two main casein subtypes and Cu^2+^. C) Preparation process of casein microgels. D) Illustration showing morphology change of CMG during Cu^2+^ capture. Scale bars = 100 µm. E) A working model summarizing the metabolic modulating effects of CuCMG in myocardial infarction.

In order to screen suitable Cu carriers, we chose several proteins commonly used in bioengineering as the candidates and identified them with theoretical molecular docking and experimental comparison. The Cu binding ability of seven proteins, including casein, bovine serum albumin (BSA), human serum albumin (HSA), collagen, lactoglobulin, sericin, and silk fibroin, were first studied using molecular stimulation (**Figure** **1**A). We ultimately chose casein (mainly composed of αS1‐Casein and β‐Casein), which had the highest Cu loading capacity, as the carrier material (Figure 1B; Figure S1, Supporting Information). Casein is the main protein (≈80%) in milk, which has extensive resources and great quality control. Casein has also shown great biocompatibility, low immunogenicity, and a relatively long degradation period (>35 days) in vivo,^[^ ^44^ ^]^ which endow it with long‐term intracorporal drug release. To facilitate intramyocardial injection, we prepared casein microgels (CMGs) by Ru/SPS redox system (Figure 1C). Cu^2+^ loading and release capacity of casein (Figure 1D), and Cu^2+^ induced structural and mechanical changes of CMGs were further studied to reflect the interaction between Cu and casein. The physiological functions of Cu^2+^ loaded CMGs (CuCMGs) on cardiacmyocytes, fibroblasts, and endothelial cells, which play important roles in myocardial remodeling were studied in vitro to evaluate the effects of CuCMGs on cell protection in a pathologic environment. The therapeutic effects and metabolic modulating functions of CuCMGs were studied in a rat acute MI model. A whole genome sequencing was proceeded to identify the important pathways CuCMGs participated in post‐MI treatment. Emphasized metabolic modulation, we further analyzed the effects of CuCMGs on leveraging the electron transport chain (ETC) functions of mitochondria in cardiac tissues. In this study, we created an intramyocardial injection microgel, CuCMG, which features in maintaining metabolic homeostasis after MI for cardiac protection, and provided a new insight into developing cardiac protecting biomaterials (Figure 1E).

## 2 Results

### 2.1 CMG is a Great Carrier of Cu^2+^

**Figure 2:**
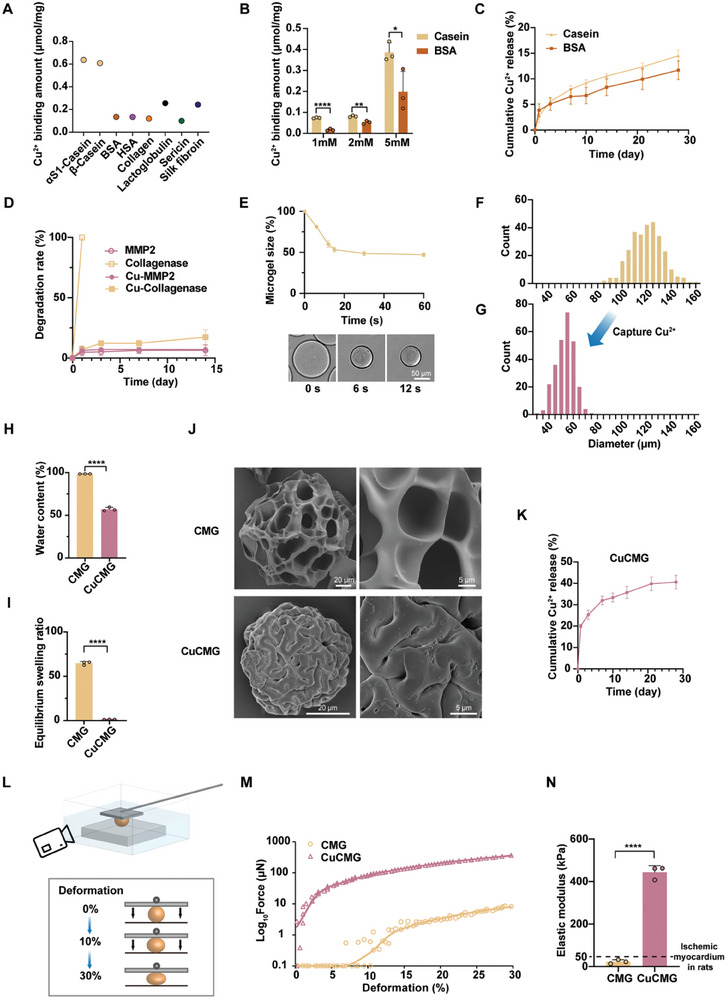
Characteristics of casein hydrogel in loading Cu. A) Molecular docking results of Cu^2+^ bound capability of widely used proteins in tissue engineering. B) Experimental results of Cu adsorption ability of casein compared to BSA. C) Release curves of Cu^2+^ from Casein hydrogel and BSA hydrogel. D) Degradation of casein hydrogel with or without Cu^2+^ in according to MMP2 or collagenase treatment, respectively. E) The size change of CMG in according to Cu^2+^ addition. F) Size distribution of CMGs and G) CuCMGs. H) Water content of CMGs and CuCMGs. I) Equilibrium swelling ratio of CMGs and CuCMGs. J) Typical scanning electron microscope images of freeze‐dried CMG and CuCMG under different magnifications. K) Cu^2+^ release curve of CuCMGs. L) Schematic of elastic modulus test of CMGs and CuCMGs. M) Typical Force‐indentation curves for CMG and CuCMG. N) The elastic moduli of CMGs and CuCMGs. The dashed line indicates the elastic modulus of ischemic myocardium in rats. Student's t‐test was used for statistical analyses. *p < 0.05, **p < 0.01, ***p < 0.001 and ****p < 0.0001. ns, no statistical significance.

To compare the Cu binding ability of different protein‐based biomaterials, molecule docking simulation was performed to sieve proteins with a high Cu^2+^/protein binding ratio. Commonly used proteins in tissue engineering, including BSA, HSA, collagen, lactoglobulin, sericin, silk fibroin, and casein, were evaluated in our study. The results showed that αS1‐Casein and β‐Casein, the most significant subtypes in bovine casein showed superior Cu^2+^ binding capacity. The Cu^2+^ binding amount of αS1‐Casein was 0.64 µmol mg^−1^, and the Cu^2+^ binding amount of β‐Casein was 0.61 µmol mg^−1^, both of which were more than twice that of other proteins (**Figure** **2**A). In order to further clarify the actual Cu^2+^ binding ability of casein hydrogel, 10% casein and 10% BSA were cross–linked by Ru/SPS redox system, and the adsorption of Cu^2+^ was measured experimentally. As the concentration of Cu^2+^ stock solution increased from 1 to 5 mm, the Cu^2+^ binding amount into the hydrogels increased dramatically. Nevertheless, in all cases, casein hydrogel had significantly higher Cu^2+^ uptake (over 150% higher) than BSA hydrogel (Figure 2B). The results indicated that casein hydrogel could be a great candidate for Cu^2+^ delivery because of its high loading capacity. Cu release of casein and BSA were also compared with the same Cu^2+^ load (different protein amount). Both casein hydrogels and BSA hydrogels showed slow and continuous Cu^2+^ release, with only 10% Cu^2+^ released after 4 weeks (Figure 2C). After Cu loading, the degradation rate of casein in response to enzymes was altered (Figure 2D). In the case of collagenase, untreated casein hydrogel was completely decomposed on the first day, but only 17.39% ± 5.00% of Cu‐loaded casein degraded after 14 days. As casein lacks matrix metalloproteinase 2 (MMP2) cleavage sites, MMP2 showed weak degradability to casein regardless of Cu loading. The results indicated that casein hydrogel had the potential to serve as a Cu sustained‐release reservoir regardless of the physiological environment rich in protease.

Casein microgels (CMGs) were prepared by an emulsification photopolymerization method and used as a Cu^2+^ carrier for in vitro and in vivo experiments. We found that CMGs retained the great Cu^2+^ binding ability of casein hydrogel, with 0.32 ± 0.02 µmol mg^−1^ Cu^2+^ loaded in CMG from a 2 mm Cu^2+^ stock solution, which is even higher than bulk casein hydrogel. Interestingly, we observed an ultrafast (within 20 s, Figure 2E) and dramatic (nearly 50%, Figure 2F,G) size reduction upon Cu^2+^ addition of CMGs (Video S1, Supporting Information). The water content of CMGs also significantly decreased from 98.48% ± 0.04% to 57.05% ± 1.80% (Figure 2H). Meanwhile, the equilibrium swelling ratio of CMGs decreased from 64.75 ± 1.55 to 1.33 ± 0.10 (Figure 2I). Scanning electron microscopy (SEM) showed that the surface microstructure of CuCMG was smoother than CMG as the original porous structure of the freeze‐dried CuCMGs disappeared (Figure 2J). Cross–linking density calculations revealed significant changes in CMG before and after Cu binding, with two orders of magnitude increase from 10^−6^ to 10^−4^ mol cm^−3^. Meanwhile, the circular dichroism (CD) spectrum showed that after cross–linking with the Ru/SPS system, the peak at 200 nm in the original casein shifted toward 240 nm, indicating that the cross–linking process affected the structure of the protein. This 240 nm peak disappeared after CMG binding Cu^2+^, suggesting Cu^2+^ further altered the protein structure (Figure S2, Supporting Information). These structural changes were also reflected in the differential scanning calorimetry (DSC) thermograms, where the denaturation temperature (*T*_m_) of CMG decreased relative to casein and increased after the adsorption of Cu^2+^. Alterations in the peak area further demonstrated structural changes with microgel preparation and Cu binding. The peak at 50 nm provided additional evidence of these changes (Figure S3, Supporting Information). Besides, we found that the conductivity of casein hydrogel increased from 0.050 ± 0.033 to 0.287 ± 0.033 S cm^−1^ after loading Cu^2+^, which might be due to the movement of metal ions (Figure S4, Supporting Information), but the value is still significantly lower than that of normal biological tissues (30–70 S cm^−1^).^[^ ^45^ ^]^ Being fabricated to microgels, the CuCMGs showed faster Cu^2+^ release than bulk gels due to the higher specific surface area but still showed a sustained release, with nearly 40% released after 4 weeks (Figure 2K). Cu loading also significantly increases the elastic modulus of CMGs. By recording the sphere deformation and the repelling force using a cantilever (Figure 2L), the force‐indentation curves of CMGs and CuCMGs were obtained (Figure 2M). The elastic moduli of CMGs were 22.5 ± 7.9 kPa calculated from the force‐indentation curves, while the elastic moduli of CuCMGs significantly increased ≈20 times to 443.4 ± 25.8 kPa (Figure 2N). Therefore, the above results all indicated that CMG was an excellent Cu‐loaded material, and after capturing Cu^2+^, the microgels became denser and more rigid.

### 2.2 CuCMGs Protect Cells in Cardiac Tissues under Pathological Environments

**Figure 3:**
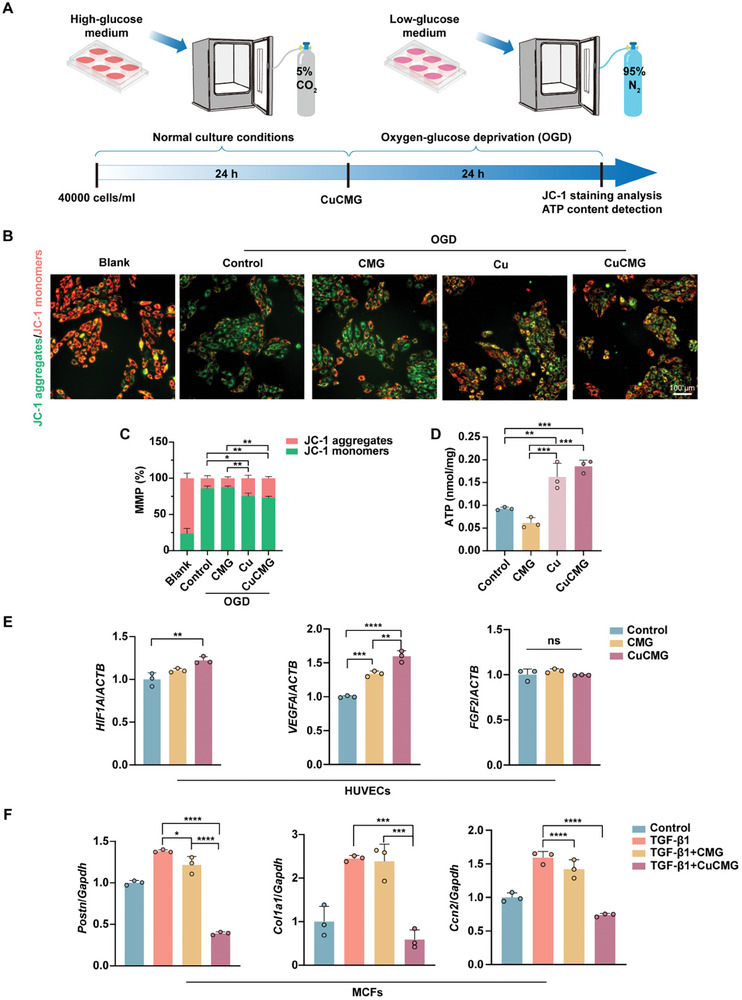
The effects of CuCMG on cardiomyocytes, endothelial cells, and fibroblasts. A) Schematic diagram of OGD model building, dosing, and detection times. B) JC‐1 staining of H9C2 cells from Blank, Control, CMG, Cu, and CuCMG groups. *n =* 4. Scale bars = 100 µm. C) Statistical analysis of the JC‐1 staining. D) The ATP levels of H9C2 cells in different groups after OGD treatment. E) qRT‐PCR analysis of the expression levels of *HIF1A*, *VEGFA*, and *FGF2* in HUVECs treated with CMG and CuCMG separately. F) qRT‐PCR analysis of the expression levels of *Postn*, *Col1a1*, and *Ccn2* in pre‐induced MCFs treated with CMG and CuCMG separately. one‐way ANOVA was used for statistical analyses. *p < 0.05, **p < 0.01, ***p < 0.001 and ****p < 0.0001. ns, no statistical significance.

The rescue of mitochondrial function in injured cardiomyocytes is critical to repair infarcted myocardium.^[^ ^46^ ^]^ To determine the role of Cu^2+^ in preserving mitochondrial functions in pathological environments in an ischemic heart, we tested whether CuCMG could protect the mitochondria of H9C2 cells (rat cardiomyocytes) from oxygen‐glucose deprivation (OGD) injury. The safe concentration range of Cu^2+^ was first determined by live/dead staining and CCK8 assay (Figures S5 and S6, Supporting Information). A timeline of the in vitro experimental design is presented in **Figure** **3**A. Only when the Cu^2+^ concentration was lower than 20 µm, the cell viability was not affected in both normoxia and OGD conditions. Thus, a Cu^2+^ concentration of 20 µm was chosen for subsequent cell experiments, and the CuCMGs concentration was adjusted to 2 mg mL^−1^ accordingly so that the Cu^2+^ concentration released within 24 h was also ≈20 µm. Mitochondria proceed OXPHOS through membrane potential gradients generated by electron transfer chains (ETCs), thereby driving adenosine triphosphate (ATP) synthesis. Therefore, mitochondrial membrane potential (MMP) is one of the important indicators for evaluating normal mitochondrial function.^[^ ^47^ ^]^ JC‐1 molecules form aggregates and show red fluorescence in healthy mitochondria, and as the MMP decreases, JC‐1 becomes monomers and shows green fluorescence. The MMP was evaluated in OGD‐treated H9C2 cells by JC‐1 staining. Figure 3B showed that both dissociative Cu^2+^ and CuCMGs treatment effectively improved the MMP in OGD‐treated H9C2 cells, as evidenced by the shift in fluorescence from green to red. The quantitative analysis further confirmed these results, that the proportion of aggregates with red fluorescence increased from 14.08% ± 3.03% in the control group to 27.10% ± 2.00% in the CuCMG group (Figure 3C). In addition, to explore whether CuCMGs treatment could increase intracellular ATP content in OGD‐treated H9C2 cells, we measured the intracellular ATP levels of different groups by chemiluminescence method. CuCMGs significantly rescued the ATP content of H9C2 cells impaired by OGD treatment (Figure 3D). In summary, CuCMGs maintained mitochondrial function in OGD conditions.

Cu^2+^ has been recognized as an effective angiogenesis stimulator, which affects many processes including endothelial cell proliferation, migration, and angiogenesis.^[^ ^48^, ^49^ ^]^ In addition, studies have shown that Cu^2+^ could reduce cardiac fibrosis after MI by inhibiting the transformation of fibroblasts into myofibroblasts.^[^ ^30^ ^]^ In our experiment, we also found that Cu^2+^ treatment within a certain concentration range could effectively promote the tube formation of human umbilical vein endothelial cells (HUVECs) and the production of vascular endothelial growth factor (VEGF) (Figures S7 and S8, Supporting Information). Also, we evaluated the function of CuCMGs on HUVECs and found that CuCMG promoted tube formation by 76.46% with the expression of vascular‐related genes (*HIF1A*, *VEGFA*, and *FGF2*) in HUVECs promoted (Figure 3E; Figure S9, Supporting Information). Moreover, when evaluating the function of myocardial fibroblasts (MCFs) activated by transforming growth factor‐β1 (TGF‐β1), we found that the expression of fibrosis‐related genes (*Postn*, *Col1a1*, and *Ccn2*) in MCFs were inhibited (Figure 3F).

### 2.3 CuCMGs Protect the Heart from Ischemic Injury

**Figure 4:**
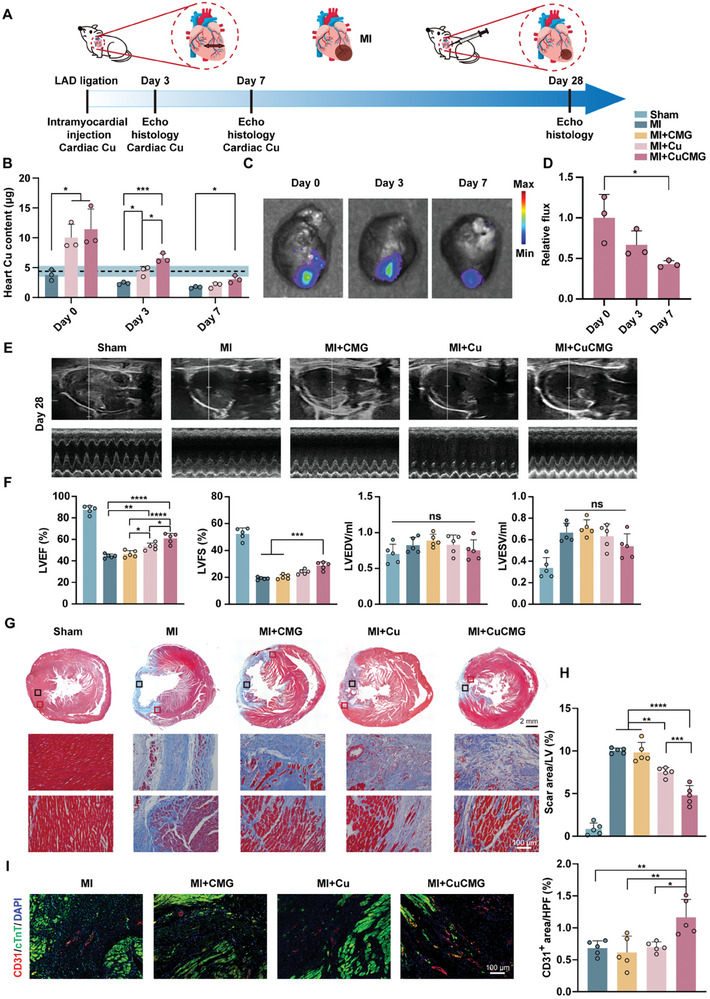
Therapeutic effects of CuCMG in treating myocardial infarction. A) Schematic diagram of MI model and CuCMG administration in rats. B) Cardiac Cu^2+^ levels at different time points after MI. C) Ex vivo imaging and D) fluorescence intensity analysis of Cy‐5 labeled CuCMGs in rat hearts. E) Representative echocardiography obtained from the middle area of the left ventricular papillary muscle in rats on Day 28 after MI. F) Echocardiography analysis of LVEF, LVFS, left ventricular end‐diastolic volume (LVEDV) and left ventricular end‐systolic volume (LVESV) at Day 28 after injection. G) Masson's trichrome staining of rat hearts in Sham, MI, MI+CMG, MI+Cu, and MI+CuCMG groups (first panel) and enlarged images of the infarcted zone (middle panel, enlarged from the black box of the first panel) and border zone (lower panel, enlarged from the red box of the first panel). H) Quantitative analysis of scar area. I) Images and quantification of CD31^+^ area in the border zone of infarcted hearts on Day 28 after MI. Scale bars = 100 µm. HPF, area of high‐power field. one‐way ANOVA was used for statistical analyses. *p < 0.05, **p < 0.01, ***p < 0.001 and ****p < 0.0001. ns, no statistical significance.

Due to the decrease in local Cu^2+^ content after MI, we hypothesized that the introduced CuCMGs could serve as a Cu^2+^ reservoir to maintain local Cu^2+^ levels and protect myocardial function in the ischemic heart. Rat MI model proceeded to verify this hypothesis, the schematic diagram is shown in **Figure** **4**A. We first detected the Cu^2+^ supplementation ability of CuCMGs in the myocardium, quantitative results showed that the amount of Cu^2+^ in the ischemic heart decreased after MI, and the treatment of CuCMGs elevated the local Cu^2+^ content for at least 7 days, while the increase in Cu^2+^ content caused by direct Cu^2+^ injection regressed to the level of the MI at Day 7 (Figure 4B). To explore the retention of microgels, CY5 labeled CuCMGs in the heart were traced with a multimodal animal live imaging system at different time points after MI. ≈50% fluorescence intensity remained on Day 7, indicating the retention of microgels (Figure 4C,D). Cu^2+^ level in serum was also traced for evaluating the systemic toxicity of CuCMGs after intramyocardial injection, serum Cu^2+^ level in rats gradually increased after MI, intramyocardial injection of CuCMGs did not significantly change the serum Cu^2+^ level (Figure S10, Supporting Information). Besides, no significant microstructural changes were observed in the important organs (livers, spleens, kidneys, and lungs) with the introduction of CuCMGs, further indicating their good biological safety (Figure S11, supporting information). Heart function measured by echocardiograms showed no significant differences in cardiac function among different groups on Days 3 and 7 after MI (Figures S12 and S13, Supporting Information). On day 28 after MI, CuCMGs treatment significantly improved the left ventricular ejection fraction (LVEF) compared to the MI group, with LVEF values increasing from 44.44% ± 1.70% to 60.93% ± 4.22%. Meanwhile, CuCMGs treatment also increased the left ventricular fractional shortening (LVFS) from 19.16% ± 0.89% in the MI group to 28.67% ± 2.56% (Figure 4E,F). These data indicated the therapeutic benefit of CuCMGs in improving heart function after MI injury. Masson's trichrome staining analysis showed that, except for the sham surgery group, all rats showed myocardial fibrosis on Day 28 after MI, while the scar area in the CuCMG treatment group was smaller than that in MI and MI+CMG groups, with the scar area decreased by nearly 50% (Figure 4G,H). In addition, the group treated solely with Cu^2+^ also showed a smaller scar area compared to the MI and MI+CMG groups, even though the scar area in this group was still significantly larger than that in the CuCMG group. The angiogenesis after MI on Day 28 was also examined by CD31 (vascular endothelial cell marker) immunofluorescence staining. More CD31^+^ positive area was observed in the border zone of the ischemic hearts of the MI+CuCMG group as compared with those in the MI and MI+CMG groups, with CD31^+^ area increasing from 0.68% ± 0.10% in the MI group to 1.16% ± 0.25% (Figure 4I), which indicated better prognosis after blood supply occlusion.

**Figure 5:**
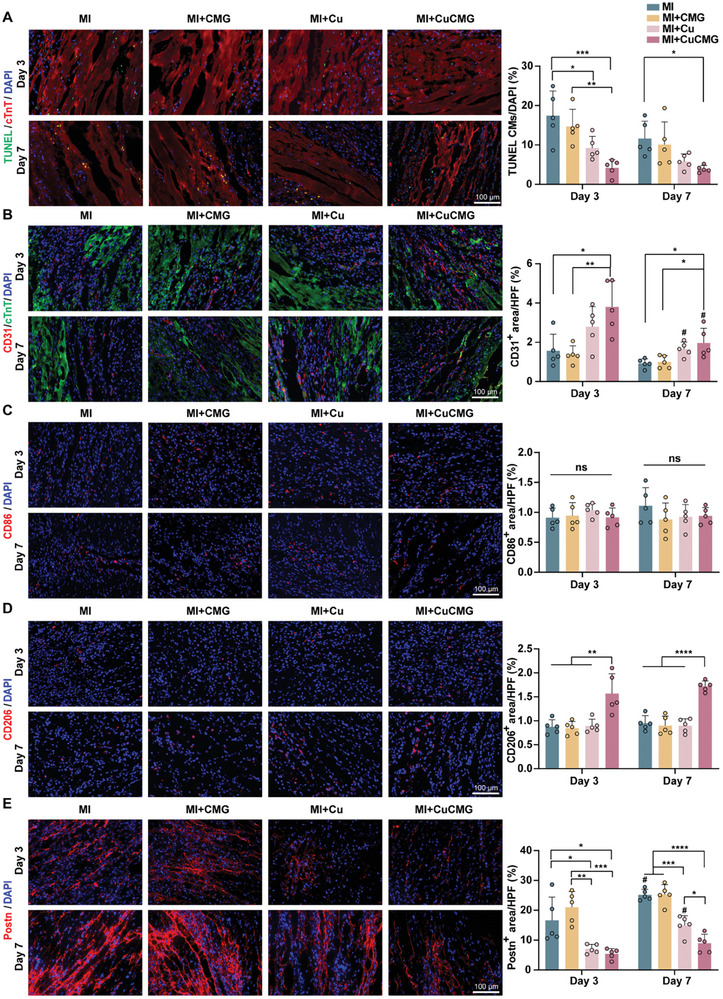
The effects of CuCMG in apoptosis, angiogenesis, inflammation, and fibrosis in the early stage after myocardial infarction. Typical images and quantification of A) TUNEL, B) CD31, C) CD86, D) CD206, and E) Postn staining showing the border zone of infarcted hearts at Day 3 and Day 7 after MI. Scale bars = 100 µm. HPF, area of high‐power field. one‐way ANOVA was used for statistical analyses. *p < 0.05, **p < 0.01, ***p < 0.001 and ****p < 0.0001. ns, no statistical significance. # *p <*0.05 compared to Day 3.

We further conducted multiple histological analyses to evaluate CuCMGs’ effect on different cell types at the early stage after MI, which may affect the late stage cardiac functions. CuCMGs treatment significantly decreased the ratio of TUNEL^+^ apoptotic cells by 75.80% and 66.38% compared to MI group on Day 3 and Day 7, respectively (**Figure** **5**A). The treatment of CuCMGs significantly increased angiogenesis examined by CD31 for about twice at different time points (Figure 5B). The CD31^+^ area decreased from Day 3 to Day 28, which is consistent with previous reports as most capillary endothelial cells in the border zone cease to proliferate in the mid to late stage of MI.^[^ ^50^, ^51^ ^]^ The polarization of macrophages was analyzed by CD86 (pro‐inflammatory phenotype marker) and CD206 (anti‐inflammatory phenotype marker) staining (Figure 5C,D). CuCMGs did not reduce the number of CD86^+^ cells but significantly increased the number of CD206^+^ cells, indicating the anti‐inflammatory potential of CuCMGs. The major effector in MI‐induced fibrosis, periostin (Postn) was stained to evaluate the response of fibroblasts. From Day 3 to Day 7 after MI, Postn^+^ area increased from 16.62% ± 6.98% to 25.15% ± 1.63%. CuCMGs or Cu^2+^ treatment ameliorated the process, with significant differences between the two groups observed on Day 7 (Figure 5E), indicating the beneficial effect of controlled release of Cu^2+^ from microgels. Thus, the results from different cell types indicated that CuCMG showed positive effects on MI treatment.

### 2.4 CuCMGs Rescue the Transcriptome of Ischemic Hearts

**Figure 6:**
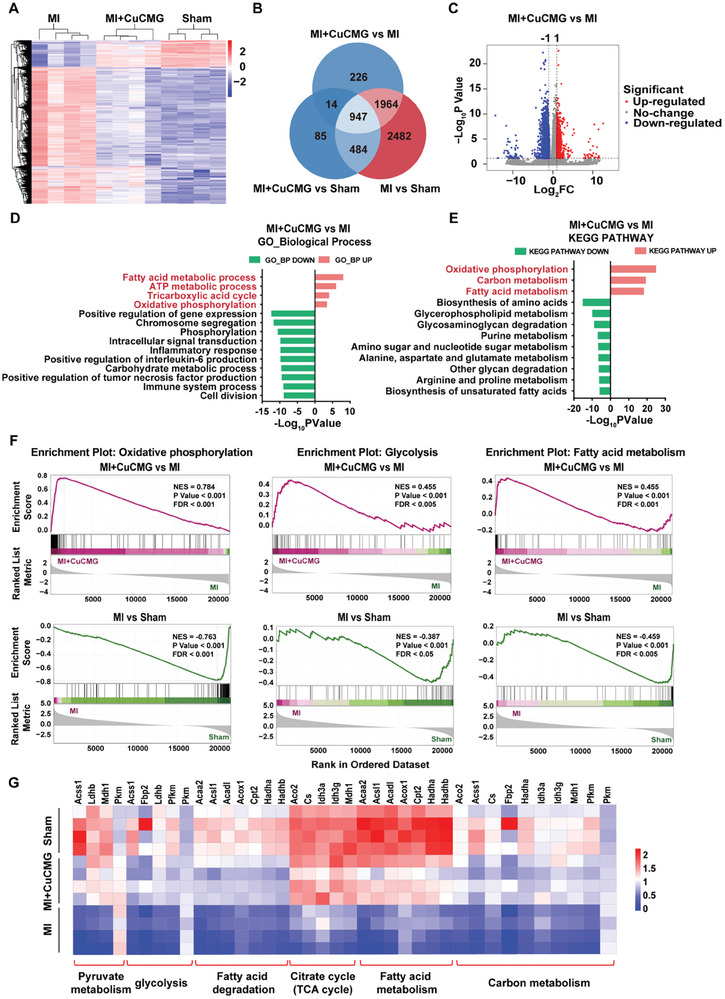
Intramyocardial injection of CuCMGs rescued transcriptomes of ischemic heart. A) Hierarchical clustering of differentially expressed genes in MI, MI+CuCMG, and Sham hearts as assessed by RNA‐seq. B) Venn diagram of differentially expressed genes in three groups. C) Volcano plot of all of the expressed genes from the MI and MI+CuCMG group. (|Log_2_Fold Change| ≥1, adjusted *p*‐Value < 0.05). D) GO analysis linked to the biological process of differentially expressed genes in MI+CuCMG hearts and MI hearts. E) KEGG pathway enrichment analysis of differentially expressed genes in MI+CuCMG hearts and MI hearts. F) GSEA analysis of metabolic‐related enrichment plots compared to MI group. G) Heat maps of metabolic‐related genes.

To clarify the comprehensive roles of CuCMGs in mediating cardiac protection following MI, we performed RNA sequencing (RNA‐seq) on the ventricular tissues of Sham, MI, MI+CMG, and MI+CuCMG hearts on Day 3. Firstly, principal component analysis (PCA) confirmed the consistency between each group of biological repeats (*n =* 4; Figure S14A, Supporting Information). Hierarchical clustering analysis showed that CuCMGs local injection significantly altered the cardiac transcriptome after MI (**Figure** **6**A). Venn diagram revealed that compared to the Sham group, MI injury altered the expression of 5877 genes in rats, of which 33.42% (1964 genes) changes could be fully restored to the level before MI by local injection of CuCMGs (Figure 6B). After that, we found 3151 differentially expressed genes between the MI+CuCMG group and the MI group, of which 687 genes were upregulated and 2464 genes were downregulated (Figure 6C). Gene Ontology (GO) biological process analyses showed that the “fatty acid metabolic process”, “ATP metabolic process”, “tricarboxylic acid cycle” and “oxidative phosphorylation” were the most enriched in upregulated genes (Figure 6D). Similar to the above description, Kyoto Encyclopedia of Genes and Genomes (KEGG) pathway analyses revealed that the altered genes were largely associated with energy metabolism‐related pathways, with upregulated genes concentrated in pathways such as “oxidative phosphorylation”, “carbon metabolism”, and “fatty acid metabolism” (Figure 6E). However, the number of differentially expressed genes between the MI+CMG group and the MI group was very small (Figure S14B–D, Supporting Information). This indicated that the gene expression difference caused by CuCMGs was mainly due to the release of Cu^2+^.

These findings suggested that intramyocardial injection of CuCMGs improved cardiac function after MI probably by mediating cardiac metabolism‐related pathways. To verify this hypothesis, we compared and analyzed the transcriptome characteristics between MI hearts treated with or without CuCMGs, as well as between MI hearts and Sham hearts. Gene Set Enrichment Analysis (GSEA) showed that CuCMGs significantly restored MI‐induced down‐regulation of metabolic pathways such as “oxidative phosphorylation”, “glycolysis”, and “fatty acid metabolism” as compared to MI group (Figure 6F). Further sorting out the gene expression status of each group, it was found that CuCMGs positively regulated the expression of many metabolic‐related genes after MI, including “pyruvate metabolism”, “glycolysis”, “fatty acid degradation”, “citrate cycle”, “fatty acid metabolism” and “carbon metabolism” (Figure 6G). These results supported the hypothesis that CuCMGs leverage important roles in cardiac metabolism after MI.

### 2.5 CuCMGs Improve Cardiac Metabolic Homeostasis after MI

**Figure 7:**
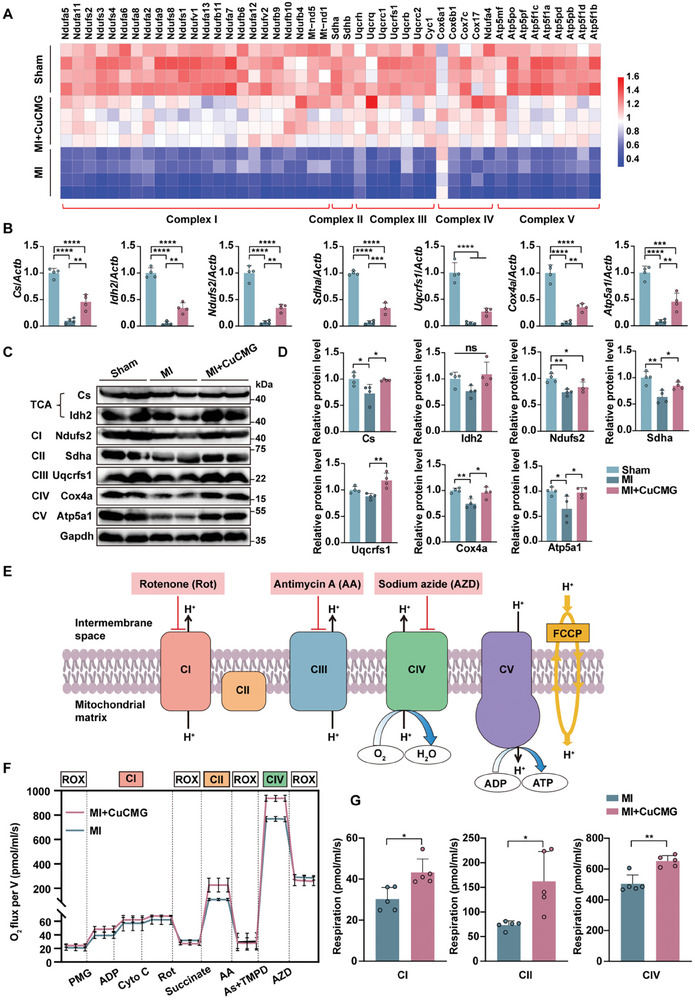
CuCMGs improved cardiac metabolic homeostasis after myocardial infarction. A) Heat maps of five enzyme complexes related genes on the electron transport chain in MI, MI+CuCMG, and Sham hearts extracted from RNA‐seq. B) qRT‐PCR analysis of the expression levels of *Cs*, *Idh2*, *Ndufs2*, *Sdha*, *Uqcrfs1*, *Cox4a*, and *Atp5a1*. C) Western blot of corresponding OXPHOS representative proteins in infarcted myocardium. D) Quantification of Western blot band intensity. E) Schematic diagram showing ETC and the represented inhibitors. Rotenone (Rot) is an inhibitor of mitochondrial complex I. Antimycin A (AA) is an inhibitor of mitochondrial complex III. Sodium azide (AZD) is an inhibitor of mitochondrial complex IV. F) Representative respiratory experiment of mitochondrial OXPHOS ability to interrogate sequentially different substrates and coupling states using myocardial filaments. G) Analysis of mitochondrial OXPHOS capacity of complex I (CI), complex II (CII), and complex IV (CIV) in ETC. Complex respiration rates were corrected to corresponding residual oxygen consumption (ROX). Student's t‐test was used for statistical analyses. *p < 0.05, **p < 0.01, ***p < 0.001 and ****p < 0.0001. ns, no statistical significance.

The results of RNA‐seq analysis revealed that CuCMGs regulated metabolic processes after MI, which prompted further investigation into how CuCMGs alleviated cardiac metabolic stress in response to heart injury and remodeling. Respiratory function is one of the most fundamental energy metabolism activities in organisms, and mitochondria are the main site for aerobic respiration in cells, providing energy for cell metabolism. The function of the respiratory chain represents the most basic function of mitochondria, and the mitochondrial respiratory chain enzyme complexes ultimately form ATP through a series of redox processes, providing energy for the body's tissues.^[^ ^52^, ^53^ ^]^ By evaluating the five enzyme complexes related genes on the electron transport chain in the hearts of MI, MI+CuCMG, and Sham groups by RNA‐seq, we found that CuCMGs had a positive regulatory effect on the expression of mitochondrial respiratory‐related genes after MI (**Figure** **7**A). To validate these results, we performed qRT‐PCR analysis on typical signature OXPHOS‐related genes which showed significant differences in RNA‐seq, including the genes of key enzymes in TCA cycle, citrate synthase (*Cs*) and isocitrate dehydrogenase (*Idh2*),^[^ ^54^ ^]^ as well as mitochondrial complex related genes (*Ndufs2*, *Sdha*, *Uqcrfs1*, *Cox4a*, and *Atp5a1*, representing genes from complex I to V).^[^ ^55^, ^56^ ^]^ Our results confirmed the upregulation of mitochondrial respiratory‐related pathways after CuCMGs treatment (Figure 7B). Further, we proceeded with a western blot (WB) to detect the protein expression of the corresponding OXPHOS indicators mentioned above. The results agreed that intramyocardial injection of CuCMGs rescued the expression of OXPHOS‐related processes after MI in both gene and protein levels. (Figure 7C,D).

The mitochondrial ETC is located on the inner membrane of mitochondria and consists of five complexes, each with its specific substrates and inhibitors (Figure 7E). Mitochondrial respiration was evaluated by measuring the oxygen consumption rate (OCR) of the ischemic myocardial filament adjacent to the border zone of the ventricle using high‐resolution respirometry based on substrate‐uncoupler inhibitor titration (SUIT) protocols.^[^ ^11^, ^57^, ^58^ ^]^ A comprehensive analysis was conducted on the OXPHOS ability of each complex in mitochondria and was evaluated by monitoring OCR while sequentially adding corresponding substrates and inhibitors. (Figure 7F). Compared with the MI group, in the presence of adenosine diphosphate (ADP) and cytochrome C (Cyto C), the respiration of complex I in the MI+CuCMG hearts significantly increased to 143% by adding substrates pyruvate, malate, and glutamate (PMG). The respiration of complex II in the MI+CuCMG group increased to 143% of the MI group in the presence of substrate succinate by adding ascorbate (As) and tetramethyl‐phenylenediamine (TMPD) as substrates, the respiration of complex IV in the MI+CuCMG hearts increased to 129% compared to the MI hearts (Figure 7G). In summary, these results indicated that CuCMGs improved cardiac OXPHOS, maintained cardiac metabolic homeostasis, and improved cardiac function after MI.

## 3 Discussion

Myocardial ischemia is mainly caused by coronary artery thrombosis. Due to the high incidence rate and mortality, it poses a huge threat to public health.^[^ ^59^ ^]^ In the infarcted heart, the respiratory and OXPHOS functions of myocardial mitochondria, which are key organelles of Cu metabolism, are significantly impaired, affecting overall metabolic levels and ultimately damaging systolic function.^[^ ^60^ ^]^ In this study, we used casein as a carrier to construct Cu‐loaded microgels (CuCMGs) that could be injected into the myocardium. Through in vitro cell experiments and in vivo rat MI models, we investigated the cellular protective and metabolic regulatory effects of CuCMGs in the pathological environment of ischemia and hypoxia. We found that CuCMGs indeed have the function of maintaining metabolic homeostasis after MI to protect cardiac function. Casein, as a protein derived from food sources, not only has lower costs compared to other natural sources of proteins, but molecular simulation docking results also demonstrated superior Cu^2+^ loading capacity. Therefore, we used the Ru/SPS redox system to construct casein microgels (CMGs) through visible light cross–linking. In the process of CMG loaded with Cu^2+^, we observed a rapid size decrease, accompanied by significant increase in cross–linking density and elastic modulus. Combined with the changes in CD spectra and DSC thermograms, we anticipated structural change of protein in CMG after Cu^2+^ loading. The structural change condensed the microgels and affected the exposure of related sites, which further affected the sensitivity to collagenase, resulting in slower degradation. As the elastic modulus of ischemic myocardium increased significantly compared to normal myocardium, from 10–20 to ≈50 kPa,^[^ ^61^ ^]^ soft microgel may be distorted under compression. The elastic modulus of CuCMG was significantly higher than that of ischemic myocardial tissue, indicating that the degree of deformation of CuCMG during cardiac contraction could be ignored after intramyocardial injection, which was beneficial in eliminating squeeze‐induced Cu release. Therefore, CuCMG was a good carrier for achieving precise delivery and sustained release of Cu^2+^ to cardiac tissue.

The dysfunction and apoptosis of cardiomyocytes, loss of vascular, and activation of fibroblasts play important roles in myocardial remodeling after ischemia.^[^ ^10^, ^62^ ^]^ We thus studied the function of CuCMGs in rescuing the mitochondrial function of cardiomyocytes, promoting endothelial cell angiogenesis, and inhibiting fibrotic expression of fibroblasts. In vivo experiments using rat MI models also showed that CuCMGs significantly alleviated the deterioration of cardiac function after ischemia, which could contribute to cardiomyocyte protection, angiogenesis improvement, or fibrosis inhibition. In order to determine the main pathways in which CuCMGs participated in post‐MI, we performed whole genome sequencing on the ischemic myocardium of rats. The results of RNA‐seq suggested that intramyocardial injection of CuCMGs improved cardiac function after MI mainly by mediating pathways related to cardiac energy metabolism, particularly affecting pathways related to the tricarboxylic acid (TCA) cycle, OXPHOS, and fatty acid metabolism. As the center for oxidative metabolism in eukaryotes, mitochondria are the sites for the TCA cycle and OXPHOS.^[^ ^63^ ^]^ In mitochondria, the TCA cycle and OXPHOS are closely coordinated. The nicotinamide adenine dinucleotide (NADH) and flavin adenine dinucleotide (FADH2) produced by a TCA cycle are consumed by Complex I (NADH dehydrogenase) and Complex II (succinate dehydrogenase, SDH) of ETC, respectively. Complex I and Complex II transfer electrons through ETC and ultimately produce ATP through OXPHOS. ETC is necessary for maintaining the TCA cycle, and it closely links the TCA cycle with OXPHOS.^[^ ^64^ ^]^ By further analyzing the results of RNA‐seq and validating key genes with qRT‐PCR, we observed a significant upregulation of complex‐related gene expression on ETC compared to the MI group after CuCMGs treatment. These changes have also been observed at the protein levels, as the expression of Cs (the enzyme initiates the TCA cycle) and Idh2 (the enzyme generates NADH) increased significantly. The representative proteins in mitochondrial complex I to V also showed the same trend as their corresponding genes. Based on the above results, we further focused on the functional changes of ETC.

ETC is located on the inner membrane of mitochondria and consists of five enzyme complexes.^[^ ^65^ ^]^ In addition to Complex I and Complex II mentioned earlier, Complex III, known as cytochrome c reductase, can transfer electrons to cytochrome c. Complex IV, called Cco, is the last protein complex in ETC. It carries the final reaction of ETC, transferring electrons to oxygen while expelling protons into the mitochondrial membrane gap. Protons ultimately pass through Complex V (mitochondrial ATP synthase) to synthesize ATP.^[^ ^66^ ^]^ In respiratory experiments for mitochondrial OXPHOS ability following SUIT protocol, the addition of an inhibitor of a certain complex blocks the preceding ETC flow, thus Complex III inhibitor could be used to evaluate OCR change caused by complex II. Research has found that many enzyme complexes are mediated by Cu^2+^ during their functional processes. Michael O Isei et al. found that Cu^2+^ acted on multiple sites in ETC and could regulate the flavin sites of Complex I and Complex II in a concentration‐dependent manner. Cu^2+^ also mediated the external ubiquinone binding sites of Complex III, affecting the generation of reactive oxygen species (ROS).^[^ ^67^ ^]^ In addition, copper sulfate (CuSO_4_) pretreatment counteracted the activity damage in mitochondrial Complexes I, II, IV, and V caused by 1‐methyl‐4‐phenylpyridinium (MPP^+^) in rat striatum.^[^ ^68^ ^]^ In the mitochondria of inflammatory macrophages, NADH can be rapidly consumed to produce NAD^+^ in the presence of Cu^2+^, and this process is mainly involved by Complex I.^[^ ^10^ ^]^ In addition, Complex IV uses Cu^2+^ as a cofactor and has multiple Cu^2+^ binding sites.^[^ ^69^ ^]^ Therefore, we speculated that the Cu^2+^ released by CuCMGs might intervene in the function of the entire ETC by affecting the structure and function of various complexes, thereby maintaining metabolic homeostasis after MI.

Present as a great carrier for Cu^2+^ delivery, CuCMGs show promising capability in maintaining overall ETC functions and metabolic homeostasis after MI. In the future, the weight functions of specific pathways can be further explored to unveil the key mechanism in CuCMG‐related metabolism regulation. It may also combine with other therapeutic strategies such as mechanical support, conductivity improvement, immunomodulation, etc., to further promote cardiac repair after MI.

## 4 Conclusion

In summary, more and more studies have been focused on preserving metabolic homeostasis after MI therapy. In this research, we developed a metabolism‐modulating system for ischemic myocardium by simply loading Cu to CMGs. Casein was screened out from the seven most commonly used protein materials due to its great binding capacity to Cu by combining molecule docking and experimental measurement. The Cu loading significantly diminished the size, and increased anti‐enzymolysis and mechanical modulus of CMGs, indicating a strong affinity between casein and Cu. The formed CuCMG has shown long‐acting Cu release and ensured the stability of MMP and ATP synthesis in cardiomyocytes under OGD environment. In addition, CuCMGs led to favorable changes in transcriptome levels related to energy metabolism and cardiac function. These findings confirm the rationale that biomaterials targeting metabolic homeostasis possess great potential in cardiac reconstruction, CuCMGs present as a new therapeutic agent for cardioprotection by modulating the malfunction metabolism in ischemic hearts.

## 5 Experimental Section

### Molecular Docking Method (MDM)

AlphaFold2 was used to construct the full‐length structures of the proteins and served them as the initial structures.^[^ ^70^ ^]^ Using Cu^2+^ as a ligand, the molecular structure was optimized by the Molecular Orbital PACkage (MOPAC) program and calculated the PM3 atomic charge for subsequent molecular docking.^[^ ^71^, ^72^ ^]^ AutoDock Tools 1.5.6 was used to process the initial protein structures and ligand structure, generating corresponding pdbqt files for docking.^[^ ^73^ ^]^ Docking was performed with AutoDock 4.2.6 using a 100 × 100 × 100 grid box centered on the protein binding site and 100 docking runs. To relieve unrealistic atomic contacts, energy optimization was done using Amber14 in two steps: 1000 cycles of steepest descent, followed by 500 cycles of conjugate gradient.

### Synthesis of CMGs and CuCMGs

CMGs were prepared by an emulsification photocross–linking method described as follows. The aqueous phase was prepared by dissolving SPS (Sinopharm Chemical Reagent Co., Ltd., Shanghai, China), tris(2,2‐bipyridyl) dichlororuthenium (II) hexahydrate (Ru) (Xianding, Shanghai, China) and casein (Fonterra, Shanghai, China) in 0.1 m NaOH solution to obtain a 10% w/v casein solution with 80 mm SPS and 0.3 mm photoinitiator Ru. The pH was adjusted to 6.5–7.5 and 0.1% w/v Tween60 (Ruibio, Germany) was added as dispersant and mixed thoroughly. The oil phase was liquid paraffin (HUSHI, Shanghai, China), containing 0.1% w/v emulsifier Span 80 (Aladdin, Shanghai, China). The volume ratio of the aqueous phase to the oil phase was 1:10. The aqueous phase was dropped into the oil phase, fully stirred for 10 min, and then irradiated with a white LED lamp for 10 min to ensure complete gelation. The obtained solid materials were thoroughly cleaned with n‐hexane (Aladdin, Shanghai, China), acetone (Aladdin, Shanghai, China), and phosphate buffered solution (PBS) in sequence, and stored in a 4 °C refrigerator before use.

To prepare CuCMGs, the CMGs were incubated in a 2 mm CuCl_2_ solution for at least 24 h to ensure equilibrium adsorption. Before the following microgel characterization, the microgels were rinsed with deionized water to remove potential unchelated ions.

### Cu Adsorption Capacity Of Casein Hydrogel

The amount of Cu adsorption was measured by a subtraction method. Namely, 25 µL (*v*_0_) of 10% w/v protein pre‐gel solution was taken and illuminated to form complete gelation. Each hydrogel was placed in 1 mL CuCl_2_ solution for 24 h, and the variation of Cu^2+^ concentration in solution before (*c*_0_) and after adsorption (*c*) was detected with a Cu assay kit (Nanjing Jiancheng Bioengineering Institute, Nanjing, China) according to manufacturer's instructions and measured with a plate reader (Tecan, SPARK) at 600 nm wavelength. The amount of adsorbed Cu (*Q*) was calculated following:

(1) $$Q=\frac{c_{0}-c}{v_{0}*10\%}$$

### Cu Release Measurement

The following methods were used to test the release profiles of Cu^2+^. 10 mg of microgels was placed in a 1.5 mL centrifuge tube, and then 1 mL (*v*_1_) of PBS was added and placed at 37 °C. Take samples (*v_2_*) and supplement the release medium on days 1, 3, 7, 10, 14, 21, and 28, respectively. Casein hydrogels and BSA hydrogels loaded with the same amount of Cu^2+^ were also immersed in PBS using the same method as described above. The contents of Cu^2+^ (*c*_1_ to *c_7_*)in the solution were detected by a copper assay kit as above. The cumulative release rate was calculated following:

(2) $$\begin{array}{ccc} & & \mathrm{Cumu}\mathrm{lati}\mathrm{ve}\;\mathrm{rele}\mathrm{ase}\;\mathrm{rate}\\ & & \;=\frac{c_{n}*v_{1}+\left(c_{1}+c_{2}+c_{3}+\cdots +c_{n-1}\right)*v_{2}}{0.44}*100\% \end{array}$$

*c_n_* represented the Cu concentration at the nth sampling point, and the total Cu loading amount has been determined to be 0.44 µmol according to previous calculations. Finally, the corresponding cumulative release curves were plotted.

### Characterization of Microgels

A phase‐contrast optical microscope (Lecia DMIL, Germany) was used to monitor the shape and particle size of each group of microgels. Scanning electron microscopy (GeminiSEM 300, Germany) was used to detect the surface morphology of freeze‐dried microgels. Far‐UV CD spectra were acquired on a spectrometer (Chirascan V100, Applied Photophysics Ltd, China) at 25 °C using a 0.1 cm path‐length quartz cuvette. Samples were dispersed in deionized water. Spectra were recorded from 260 to 180 nm at a scan rate of 50 nm min^−1^ with a response time of 1 sec and bandwidth of 1 nm. Three scans were accumulated and averaged followed by baseline subtraction of buffer only signal. Thermograms were obtained using a TA Instruments DSC (Q20 V24.10 Build 122, USA) from −80 to 180 °C at a scan rate of 10 °C min^−1^ with a nitrogen purge gas flow of 50 mL min^−1^. An empty pan was used as a reference to obtain the instrument baseline profile. Sample data was baseline‐corrected by subtracting the reference scan. The denaturation temperature (*T*_m_) was determined from the maximum point of the endothermic peak. A certain compressive force was applied to the microgel by using the microscale mechanical testing system (Microsquisher, CellScale) to compress it by 30 µm at a speed of 2 µm s^−1^. The elastic modulus was calculated from strain–stress curve.

### Calculation of Cross–linking Density of CMG and CuCMG

Microgels were accurately weighed (mass *m*_0_) and added to 25 mL of deionized water. The vials were capped and allowed to swell at 25 °C with solvent replacement every 6 hours. Samples were periodically removed every 30 min initially, then per hour when swelling slowed, blotted dry, and weighed (mass *m*_1_) until consecutive weighings differed by no more than 0.002 g, indicating swelling equilibrium was reached. Each sample was measured in duplicate. The cross–linking density (*ρ_c_*) was calculated as:

(3) $$\rho_{c}=-\frac{\ln\left(1-v_{r}\right)+v_{r}+\chi {v_{r}}^{2}}{V_{S}\left(v_{r}^{1/3}-0.5v_{r}\right)}$$

(4) $$v_{r}=\frac{m_{0}/\rho_{0}}{m_{0}/\rho_{0}+\left(m_{1}-m_{0}\right)/\rho_{1}}$$

Here *V_S_* was solvent molar volume, χ was Flory–Huggins polymer‐solvent interaction parameter, *ρ*_0_, and *ρ*_1_ were densities of the microgels and solvent, and *m*_0_, *m*_1_ were masses of the sample before swelling, and the swollen samples, respectively.

### Water content and Equilibrium Swelling Ratio of Microgels

The wet mass (*M_S_*) of microgels in each group was measured. The microgels were then dried in the freeze‐drying machine for 24 h and their dry mass (*M_D_*) was measured. The water content and equilibrium swelling ratio were calculated as follows:

(5) $$\mathrm{Water}\;\mathrm{cont}\mathrm{ent}=\frac{M_{S}-M_{D}}{M_{S}}*100\%$$

(6) $$\mathrm{Equi}\mathrm{libr}\mathrm{ium}\;\mathrm{swel}\mathrm{ling}\;\mathrm{ratio}=\frac{M_{S}-M_{D}}{M_{D}}$$

### Degradation of Casein Hydrogel With or Without Cu^2+^

Casein hydrogels with or without Cu treatment were put into 10 nm MMP2 (Solarbio, Beijing, China) or 1 mg mL^−1^ collagenase (Gibco, USA) respectively. The initial dry weight (*M*_0_) and the dry weight at different time points (*M_n_*) of hydrogels in each group were measured. The degradation rate was calculated as follows:

(7) $$\mathrm{Degr}\mathrm{adat}\mathrm{ion}\;\mathrm{rate}=\frac{M_{0}-M_{n}}{M_{0}}*100\%$$

### Cell Culture

H9C2 cells (rat cardiomyocytes) were purchased from Procell (Wuhan, China). The cells were cultured in high‐glucose Dulbecco's modification of Eagle medium (DMEM; Gibco, USA) supplemented with 10% fetal bovine serum (FBS; Gibco, USA) and 1% penicillin/streptomycin (P/S; Gibco, USA) in a carbon dioxide incubator (37 °C and 5% CO_2_). The OGD model was established with low‐glucose DMEM supplemented with 2% FBS, and the cells were incubated in 95% N_2_ and 5% CO_2_ for 24 h. Immortalized and green fluorescent protein (GFP) transfected human umbilical vein endothelial cells (HUVECs) were purchased from iCell (Shanghai, China) and cultured in high‐glucose DMEM supplemented with 10% FBS and 1% P/S. Isolation of myocardial fibroblasts (MCFs) from neonatal mice was performed following literature using a Neonatal Cardiac Fibroblast Isolation Kit (Miltenyi, Germany).^[^ ^74^ ^]^ MCFs were cultured with high‐glucose DMEM supplemented with 5% FBS and 1% P/S.

### JC‐1 Staining Analysis

H9C2 cells were allowed to grow with an initial density of 40 000 cells mL^−1^ in 96‐well plates in complete DMEM for 24 h. After OGD treatment for 24 h, the original culture medium was replaced with fresh medium with 2 µm JC‐1 (MedChemExpress, USA) The plates were incubated at 37 °C for 15–20 min, washed twice with PBS, and observed under fluorescence microscopy (Leica DMi8, Germany).

### ATP Content Detection

H9C2 cells were allowed to grow for 24 h in a 12‐well plate as previously mentioned. After OGD treatment, the cellular ATP levels were measured according to the manual of the Enhanced ATP Assay Kit (Beyotime Biotechnology, Shanghai, China). ATP level was calculated according to a calibration curve with standard ATP samples. The total amount of protein in each group was detected using the BCA Protein Assay Kit (Beyotime Biotechnology, Shanghai, China) for normalization of ATP contents.

### Quantitative Reverse Transcription‑Polymerase Chain Reaction (qRT‑PCR)

Cells were lysed and RNA was extracted using a total RNA isolation kit (Vazyme Biotech, Nanjing, China). The total RNA of heart tissues was isolated using TRIzol reagent (Life Technologies). The complementary DNA (cDNA) was synthesized using a PrimeScript RT reagent kit (Accurate Biology, Hunan, China). qRT‐PCR signal was detected by the LightCycler 480 II PCR System (Roche, Switzerland) with a SYBR green qPCR SuperMix (TransGen Biotech, Beijing, China). *Gapdh* and *ACTB* were used as the housekeeping genes, respectively. ddCt was used to analyze gene expression from qPCR datasets. Primers used for qRT‑PCR in this study were purchased from Sangon Biotech and listed in Table S1 (Supporting Information).

### Myocardial Infarction

All animal experiments have been approved by the Zhejiang Experimental Animal Center (No. ZJCLA‐IACUC‐20010597). Male SD rats (8 w old, 200–250 g) were purchased from the Zhejiang Academy of Medical Sciences. The animals were anesthetized with an intraperitoneal injection of 1% w/v sodium pentobarbital and ventilated. The heart was exposed through a left‐sided thoracotomy. 6‐0 silk suture was used to ligate the LAD coronary artery to obtain left ventricular infarction. A total volume of 60 µL of 100 mg mL^−1^ CuCMGs, 100 mg mL^−1^ CMGs, or 3 mm CuCl_2_ solution was injected into the border zones of infarcted myocardium for MI+CuCMG group, MI+CMG group, and MI+Cu group, respectively. Rats only experienced a thoracotomy operation (Sham) and MI rats received no treatments (MI) were used as controls.

### Measurement of Cu^2+^ Contents in the Serum and Heart Tissue

The postoperative rats were anesthetized and blood was collected from the hearts to obtain serum. Serum Cu levels were detected with a Cu assay kit as mentioned above. Heart samples from the infarct area were obtained, and heart tissue from the same part of the heart of the Sham group was taken as controls. Heart tissue samples were freshly frozen, lyophilized, and digested with nitric acid (HNO_3_, Sinopharm, China) overnight (10 mg dried tissue with 1 mL HNO_3_). After filtration, Cu contents were determined by using a graphite furnace atomic absorption spectrophotometer (AAS, ICE3500, Thermo, USA).

### Ex vivo Fluorescence Imaging

Microgels labeled with Sulfo‐CY5‐NHS (Yusi Biotechnology Co., Ltd., Chongqing, China) were used for intramyocardial injection. The rats were euthanized to harvest their hearts at Day 0 (immediately after surgery), Day 3, and Day 7 after MI and imaged with a multimodal animal live imaging system (AniView100, BioLight, China). The excitation light wavelength was 605 nm, and the emission light wavelength was 680 nm.

### Measurement of Cardiac Function By Echocardiography

Transthoracic echocardiography (VEVO2100, Visual Sonics, Canada) was used to analyze cardiac function on Day 3, Day 7, and Day 28 after MI. Rats were anesthetized by inhaling oxygen‐containing isoflurane (1–1.5%), and their chest hair was also removed before examination. B‐mode echocardiography and M‐mode echocardiography were used for the long‐axis views for measuring cardiac parameters.

### Histological Analysis

The rats were euthanized to harvest their hearts. The hearts were soaked in potassium chloride solution to stop beating and fixed in 4% paraformaldehyde overnight at room temperature. For paraffin sectioning, heart samples were embedded in paraffin, and sections with a thickness of 6 µm were cut for following Masson's trichrome staining which was proceeded using a Modified Masson's Trichrome Stain Kit (Solarbio, Beijing, China) and observed under an optical microscopy (Leica DM3000, Germany). For cryotomy, heart samples were perfused with 30% sucrose at 4 °C overnight before being embedded in optimal cutting temperature compound (OCT) for frozen section. 6 µm sections were cut for immunofluorescence staining and TUNEL staining. Apoptotic cells were detected by TUNEL staining using the one‐step TUNEL Apoptosis Assay Kit (Beyotime, Jiangsu, China) according to the manufacturer's protocol. The immunofluorescence staining of cTnT was performed together with TUNEL staining. After blocking with PBS containing 5% w/v BSA at room temperature for 1 hour. Then, sections were incubated in a primary antibody solution Mouse anti‐cTnT (1/400 diluted in 1% BSA, abcam, USA) overnight at 4 °C. Then, sections were washed with PBS and incubated with a secondary antibody. For other immunofluorescence staining, Rabbit anti‐CD31 (1/800 diluted in 1% BSA, Servicebio, China), Rabbit anti‐CD86 (1/500 diluted in 1% BSA, Peoteintech, USA), Rabbit anti‐CD206 (1/500 diluted in 1% BSA, Peoteintech, USA), Rabbit anti‐Postn (1/500 diluted in 1% BSA, R&D Systems, USA) were used as the primary antibody with Alexa Fluor 555 donkey anti‐rabbit IgG as the secondary antibody (1/500 diluted in 1% BSA, Abcam, Britain). Finally, after incubating with DAPI (1 µg mL^−1^, Servicebio, China) at room temperature for 10 min, the sections were observed under a fluorescence microscope (Leica DM6B, Germany). The area of interest was analyzed by ImageJ software.

### RNA‑seq and Genome‑Wide Transcriptome Analysis

On Day 3 after MI, total RNA from the ventricular tissue of rats in the Sham, MI, MI+CMG, MI+Cu, and MI+CuCMG groups was collected for RNA seq (*n =* 4). RNA‐seq experiments were performed by LC‐Bio Technology (Hangzhou, China). Total RNA was extracted from fresh ventricular tissue using Trizol reagent (thermofisher) following the manufacturer's procedure. Then, mRNA was purified from total RNA using Dynabeads Oligo (dT) (Thermo Fisher, CA, USA).

A cDNA library constructed by technology from the pooled RNA from samples was sequenced and run with the Illumina Novaseq TM 6000 sequence platform. The Illumina paired‐end RNA‐seq approach was used to sequence the transcriptome, generating 150 bp paired‐end reads. Reads obtained from the sequencing machines included raw reads containing adapters or low‐quality bases which might affect the following assembly and analysis. To get high‐quality clean reads, reads were further filtered by Cutadapt. The sequence quality was verified using FastQC. The reference genome and gene model notes files were downloaded directly from the genome website.

Principal component analysis (PCA) was performed with the princomp function of R in this experience. Genes differential expression analysis was performed by DESeq2 software between two different groups. The genes with the parameter of false discovery rate (FDR) below 0.05 and absolute fold change ≥ 2 were considered differentially expressed genes. Differentially expressed genes were then subjected to enrichment analysis of GO functions and KEGG pathways. GO terms and KEGG pathways with corrected *p < 0.05* were considered significantly enriched in differentially expressed genes. We performed gene set enrichment analysis using software GSEA (v4.1.0) and MSigDB to identify whether a set of genes showed significant differences in the two groups. The hierarchical clustering heat map was performed using the OmicStudio tools.

### Western blot (WB) Analysis

Protein lysates were prepared from heart tissues using tissue extraction reagents (Invitrogen) supplemented with proteinase inhibitors. The samples were separated by 10% sodium dodecyl sulfate‐polyacrylamide gel electrophoresis (SDS–PAGE) and transferred onto polyvinylidene fluoride (PVDF) membranes by electrophoresis. After blocking in 5% skimmed milk, membranes were incubated with the indicated primary antibodies overnight at 4 °C and then washed with phosphate‐buffered saline with 0.05% Tween20 (PBST) buffer before incubation with horseradish peroxidase (HRP)‐conjugated secondary antibodies for 2 h at room temperature. Protein bands were visualized using enhanced chemiluminescence (ECL) Reagent (Invitrogen) with the Bio‐Rad ChemiDoc imaging system. Quantification of WB was analyzed by ImageJ software. All the antibody information is listed in Table S2 (Supporting Information).

### Mitochondrial Respiration in Myocardial Filaments

Ten milligrams of myocardial tissue adjacent to the border zone was dissected from each heart and separated into fiber bundles as fine as possible by sharp tweezers on ice. Then the fiber bundles were permeabilized in ice‐cold biological sample solution (BIOPS, composed of 2.77 mm CaK_2_EGTA, 7.23 mm K_2_EGTA, 5.77 mm Na_2_ATP, 6.56 mm MgCl_2_, 20 mm Taurine, 15 mm Na_2_Phosphocreatine, 20 mm Imidazole, 0.5 mm Dithiothreitol, 50 mm 2‐(4‐Morpholino) ethane sulfonic acid hydrate, pH = 7.1) containing 50 ug mL^−1^ saponin (Sigma‐Aldrich, Germany) by shaking on a shaking table for 30 min. Permeabilized fibers were washed for 10 min by agitation in ice‐cold mitochondrial respiration medium 05 (MiR05, composed of 0.5 mm EGTA, 3 mm MgCl_2_, 60 mm K‐lactobionate, 20 mm taurine, 10 mm KH_2_PO_4_, 20 mm HEPES, 110 mm sucrose, 1 g L^−1^ BSA, pH = 7), weighed, and used for respirometric measurements immediately. 4 mg of myofibers were placed into each respiratory measurement chamber containing 2 mL of MiR05, and their respiratory function was measured at 37 °C (OROBOROS Oxygraph‐2k, Innsbruck, Austria). Data acquisition and analysis were performed by using DatLab software (OROBOROS INSTRUMENTS).

### Statistics Analysis

Statistical analysis was performed using GraphPad Prism software. Unless otherwise stated, results are presented as mean value ± standard error of the means (SEM). Statistical significance between two columns was assessed by a two‐tailed Student's t‐test; for more than two columns, a one‐way Analysis of Variance (ANOVA) was used.

## Conflict of Interest

The authors declare that they have no conflict of interest.

## Author Contributions

X.H., G.T., and B.D. contributed equally to this work. The study was conceptualized by X. H., T.R, J.W., and X.L. The methodology was developed by X.H., T.R., G.T., B.D., and X.Z. The investigation involved X. Hong, B.D., X.Z., Y.G., L.Z., H.L., Q.Z., L.Z., Y.Z., D.R., and C.G. Visualization was handled by X. H. and T.R. Supervision was provided by T.R., J.W., and X.L., X.H., and T.R. were responsible for writing, while T.R., J.W., and X.L. contributed to writing, review, and editing.

## Supporting information

Supporting Information

Supplemental Video 1

## Data Availability

The data that support the findings of this study are available from the corresponding author upon reasonable request.

## References

[1] T. Vos , Lancet 2020, 396, 1204. 33069326 10.1016/S0140-6736(20)30925-9 PMC7567026

[2] H. Tada , K. K. Yeo , J. J. Li , K. Tan , J. Ako , R. Krittayaphong , R. San Tan , P. E. Aylward , C. S. P. Lam , S. H. Baek , J. Dalal , A. Fong , Y. H. Li , R. C. O'Brien , S. Y. Natalie Koh , D. J. Scherer , V. Kang , A. J. Nelson , J. Butters , S. J. Nicholls , JACC. Asia 2021, 1, 294. 36341217 10.1016/j.jacasi.2021.08.008 PMC9627888

[3] Benjamin , Circulation 2020, 141, E33.31928433

[4] O. Kwon , D. W. Park , JACC. Asia 2022, 2, 1. 36340250 10.1016/j.jacasi.2021.12.005 PMC9627845

[5] J. Ritterhoff , R. Tian , Nat. Rev. Cardiol. 2023, 20, 812. 37237146 10.1038/s41569-023-00887-x

[6] S. C. Kolwicz , S. Purohit , R. Tian , Circ. Res. 2013, 113, 603. 23948585 10.1161/CIRCRESAHA.113.302095 PMC3845521

[7] S. P. Jones , N. E. Zachara , G. A. Ngoh , B. G. Hill , Y. Teshima , A. Bhatnagar , G. W. Hart , E. Marban , Circulation 2008, 117, 1172. 18285568 10.1161/CIRCULATIONAHA.107.730515

[8] N. B. Stottrup , B. Lofgren , R. D. Birkler , J. M. Nielsen , L. Wang , C. A. Caldarone , S. B. Kristiansen , H. Contractor , M. Johannsen , H. E. Botker , T. T. Nielsen , Cardiovasc. Res. 2010, 88, 257. 20562422 10.1093/cvr/cvq205

[9] P. Li , J. Ge , H. Li , Nat. Rev. Cardiol. 2020, 17, 96. 31350538 10.1038/s41569-019-0235-9

[10] C. J. Zuurbier , L. Bertrand , C. R. Beauloye , I. Andreadou , M. Ruiz‐Meana , N. R. Jespersen , D. Kula‐Alwar , H. A. Prag , H. E. Botker , M. Dambrova , C. Montessuit , T. Kaambre , E. Liepinsh , P. S. Brookes , T. Krieg , J. Cell. Mol. Med. 2020, 24, 5937. 32384583 10.1111/jcmm.15180 PMC7294140

[11] N. Liu , M. Kataoka , Y. Wang , L. Pu , X. Dong , X. Fu , F. Zhang , F. Gao , T. Liang , J. Pei , C. Xiao , Q. Qiu , T. Hong , Q. Chen , J. Zhao , L. Zhu , J. He , X. Hu , Y. Nie , W. Zhu , H. Yu , D. B. Cowan , X. Hu , J. Wang , D. Z. Wang , J. Chen , Basic Res. Cardiol. 2021, 116, 48. 34379189 10.1007/s00395-021-00887-3 PMC8357683

[12] Z. Y. Wang , X. X. Liu , Y. F. Deng , Cell Death Differ. 2022, 29, 709. 34621049 10.1038/s41418-021-00885-x PMC8989981

[13] G. Ruozi , F. Bortolotti , A. Mura , M. Tomczyk , A. Falcione , V. Martinelli , S. Vodret , L. Braga , M. Dal Ferro , A. Cannata , L. Zentilin , G. Sinagra , S. Zacchigna , M. Giacca , Sci. Transl. Med. 2022, 14, eabo0699. 36044596 10.1126/scitranslmed.abo0699

[14] H. Shi , T. Xue , Y. Yang , C. Jiang , S. Huang , Q. Yang , D. Lei , Z. You , T. Jin , F. Wu , Q. Zhao , X. Ye , Sci. Adv. 2020, 6, eaaz3621. 32596444 10.1126/sciadv.aaz3621 PMC7299628

[15] M. L. Lugin , R. T. Lee , Y. J. Kwon , ACS Nano 2020, 14, 14262. 33073995 10.1021/acsnano.0c03850

[16] C. Ma , M. L. Kuzma , X. Bai , J. Yang , Adv. Sci. 2019, 6, 1900819. 10.1002/advs.201900819 PMC6774061 31592416

[17] X. Hong , G. Tian , Y. Zhu , T. Ren , Regen. Biomater. 2024, 11, rbad103. 38173776 10.1093/rb/rbad103 PMC10761210

[18] G. Tian , T. Ren , Eur. J. Cell Biol. 2023, 102, 151288. 36696810 10.1016/j.ejcb.2023.151288

[19] Z. Wang , J. Hu , J. Faber , J. Miszuk , H. Sun , ACS Appl. Bio. Mater. 2022, 5, 3281. 10.1021/acsabm.2c00263 PMC9296567 35737928

[20] G. Ikeda , M. R. Santoso , Y. Tada , A. M. Li , E. Vaskova , J. H. Jung , C. O'Brien , E. Egan , J. Ye , P. C. Yang , J. Am. Coll. Cardiol. 2021, 77, 1073. 33632482 10.1016/j.jacc.2020.12.060 PMC8626617

[21] P. Chen , X. Liu , C. Gu , P. Zhong , N. Song , M. Li , Z. Dai , X. Fang , Z. Liu , J. Zhang , R. Tang , S. Fan , X. Lin , Nature 2022, 612, 546. 36477541 10.1038/s41586-022-05499-y PMC9750875

[22] C. Ma , X. Tian , J. P. Kim , D. Xie , X. Ao , D. Shan , Q. Lin , M. R. Hudock , X. Bai , J. Yang , Proc. Natl. Acad. Sci. USA 2018, 115, E11741. 30478052 10.1073/pnas.1813000115 PMC6294936

[23] H. Liu , Y. Du , J. P. St‐Pierre , M. S. Berghlt , H. Autefage , J. Wang , M. Cai , G. Yang , M. M. Stevens , S. Zhang , Sci. Adv. 2020, 6, eaay7608. 32232154 10.1126/sciadv.aay7608 PMC7096169

[24] P. Tsvetkov , S. Coy , B. Petrova , M. Dreishpoon , A. Verma , M. Abdusamad , J. Rossen , L. Joesch‐Cohen , R. Humeidi , R. D. Spangler , J. K. Eaton , E. Frenkel , M. Kocak , S. M. Corsello , S. Lutsenko , N. Kanarek , S. Santagata , T. R. Golub , Science 2022, 375, 1254. 35298263 10.1126/science.abf0529 PMC9273333

[25] C. C. Zhong , T. Zhao , C. Hogstrand , F. Chen , C. C. Song , Z. Luo , J. Nutr. Biochem. 2022, 100, 108883. 34653601 10.1016/j.jnutbio.2021.108883

[26] N. M. Garza , A. B. Swaminathan , K. P. Maremanda , M. Z. Fli , V. M. Gohil , Trends Endocrinol. Metab. 2023, 34, 21. 36435678 10.1016/j.tem.2022.11.001 PMC9780195

[27] L. Zheng , P. Han , J. Liu , R. Li , W. Yin , T. Wang , W. Zhang , Y. J. Kang , Pharmacol. Ther. 2015, 148, 66. 25476109 10.1016/j.pharmthera.2014.11.014

[28] D. Ramchandani , M. Berisa , D. A. Tavarez , Z. Li , M. Miele , Y. Bai , S. B. Lee , Y. Ban , N. Dephoure , R. C. Hendrickson , S. M. Cloonan , D. Gao , J. R. Cross , L. T. Vahdat , V. Mittal , Nat. Commun. 2021, 12, 7311. 34911956 10.1038/s41467-021-27559-z PMC8674260

[29] K. Li , C. Li , Y. Xiao , T. Wang , Y. J. Kang , Exp. Biol. Med. 2018, 243, 780. 10.1177/1535370218773055 PMC5956663 29763364

[30] Y. Xiao , Q. Feng , L. Huang , X. Meng , P. Han , W. Zhang , Y. J. Kang , J. Nutr. Biochem. 2023, 111, 109180. 36240958 10.1016/j.jnutbio.2022.109180

[31] H. Xie , Y. J. Kang , Curr. Med. Chem. 2009, 16, 1304. 19355887 10.2174/092986709787846622

[32] X. Zhang , Y. Zhang , R. Zhang , X. Jiang , A. C. Midgley , Q. Liu , H. Kang , J. Wu , A. Khalique , M. Qian , D. An , J. Huang , L. Ou , Q. Zhao , J. Zhuang , X. Yan , D. Kong , X. Huang , Adv. Mater. 2022, 34, 2110352. 10.1002/adma.202110352 35107869

[33] J. Versieck , F. Barbier , A. Speecke , J. Hoste , Clin. Chem. 1975, 21, 578. 1116293

[34] Q. Li , J. Liao , K. Zhang , Z. Hu , H. Zhang , Q. Han , J. Guo , L. Hu , J. Pan , Y. Li , Y. Li , Z. Tang , J. Inorg. Biochem. 2022, 230, 111750. 35151098 10.1016/j.jinorgbio.2022.111750

[35] W. Liu , X. Zhang , X. Jiang , B. Dai , L. Zhang , Y. Zhu , Bioact. Mater. 2024, 33, 460. 38076651 10.1016/j.bioactmat.2023.10.015 PMC10697850

[36] Z. Hu , W. Cao , L. Shen , Z. Sun , K. Yu , Q. Zhu , T. Ren , L. Zhang , H. Zheng , C. Gao , Y. He , C. Guo , Y. Zhu , D. Ren , ACS Appl. Mater. Interfaces 2022, 14, 28501. 35703017 10.1021/acsami.2c02361

[37] Y. Wang , L. Guo , S. Dong , J. Cui , J. Hao , Adv. Colloid Interface Sci. 2019, 266, 1. 30776711 10.1016/j.cis.2019.01.005

[38] Q. Feng , D. Li , Q. Li , X. Cao , H. Dong , Bioact. Mater. 2022, 9, 105. 34820559 10.1016/j.bioactmat.2021.07.020 PMC8586262

[39] H. T. Shi , Z. H. Huang , T. Z. Xu , A. J. Sun , J. B. Ge , EBioMedicine 2022, 78, 103968. 35367772 10.1016/j.ebiom.2022.103968 PMC8983382

[40] S. H. Tan , S. J. Loo , Y. Gao , Z. H. Tao , L. P. Su , C. X. Wang , S. L. Zhang , Y. H. Mu , Y. H. Cui , D. Abdurrachim , W. H. Wang , J. Lalic , K. C. Lim , J. Bu , R. S. Tan , T. H. Lee , J. Zhang , L. Ye , Theranostics 2021, 11, 7879. 34335970 10.7150/thno.56757 PMC8315077

[41] Y. Wu , T. Chang , W. Chen , X. Wang , J. Li , Y. Chen , Y. Yu , Z. Shen , Q. Yu , Y. Zhang , Bioact. Mater. 2021, 6, 520. 32995677 10.1016/j.bioactmat.2020.08.031 PMC7492819

[42] R. W. Murphy , B. E. Farkas , O. G. Jones , J. Colloid Interface Sci. 2017, 505, 736. 28662476 10.1016/j.jcis.2017.06.061

[43] Y. Lei , Y. Wang , J. Shen , Z. Cai , Y. Zeng , P. Zhao , J. Liao , C. Lian , N. Hu , X. Luo , W. Cui , W. Huang , Adv. Funct. Mater. 2021, 31, 2105084.

[44] Q. Zhu , X. Zhou , Y. Zhang , D. Ye , K. Yu , W. Cao , L. Zhang , H. Zheng , Z. Sun , C. Guo , X. Hong , Y. Zhu , Y. Zhang , Y. Xiao , T. G. Valencak , T. Ren , D. Ren , Biomater. Res. 2023, 27, 6. 36737833 10.1186/s40824-023-00346-1 PMC9898936

[45] Y. Xue , X. Chen , F. Wang , J. Lin , J. Liu , Adv. Mater. 2023, 35, 2304095. 10.1002/adma.202304095 37381603

[46] C. J. A. Ramachandra , S. Hernandez‐Resendiz , G. E. Crespo‐Avilan , Y. H. Lin , D. J. Hausenloy , EBioMedicine 2020, 57, 102884. 32653860 10.1016/j.ebiom.2020.102884 PMC7355051

[47] S. Miwa , S. Kashyap , E. Chini , T. von Zglinicki , J. Clin. Invest. 2022, 132, e158447. 35775483 10.1172/JCI158447 PMC9246372

[48] G. F. Hu , J. Cell. Biochem. 1998, 69, 326. 9581871 10.1002/(sici)1097-4644(19980601)69:3<326::aid-jcb10>3.0.co;2-a

[49] Q. F. Li , X. Q. Ding , Y. J. Kang , J. Nutr. Biochem. 2014, 25, 44. 24314864 10.1016/j.jnutbio.2013.08.013

[50] X. Wu , M. R. Reboll , M. Korf‐Klingebiel , K. C. Wollert , Cardiovasc. Res. 2021, 117, 1257. 33063086 10.1093/cvr/cvaa287

[51] S. M. Deger , A. M. Hung , J. L. Gamboa , E. D. Siew , C. D. Ellis , C. Booker , F. Sha , H. Li , A. Bian , T. G. Stewart , R. Zent , W. E. Mitch , N. N. Abumrad , T. A. Ikizler , JCI Insight 2017, 2, e95185. 29202452 10.1172/jci.insight.95185 PMC5752392

[52] I. Vercellino , L. A. Sazanov , Nat. Rev. Mol. Cell. Biol. 2022, 23, 141. 34621061 10.1038/s41580-021-00415-0

[53] A. R. Fernie , F. Carrari , L. J. Sweetlove , Curr. Opin. Plant Biol. 2004, 7, 254. 15134745 10.1016/j.pbi.2004.03.007

[54] Y. Xue , X. Chen , F. Wang , J. Lin , J. Liu , Adv. Mater. 2023, 35, 2304095. 10.1002/adma.202304095 37381603

[55] K. M. Tharp , R. Higuchi‐Sanabria , G. A. Timblin , B. Ford , C. Garzon‐Coral , C. Schneider , J. M. Muncie , C. Stashko , J. R. Daniele , A. S. Moore , P. A. Frankino , S. Homentcovschi , S. S. Manoli , H. Shao , A. L. Richards , K. H. Chen , J. ten Hoeve , G. M. Ku , M. Hellerstein , D. K. Nomura , K. Saijo , J. Gestwicki , A. R. Dunn , N. J. Krogan , D. L. Swaney , A. Dillin , V. M. Weaver , Cell Metab. 2021, 33, 1322. 34019840 10.1016/j.cmet.2021.04.017 PMC8266765

[56] S. Stoldt , D. Wenzel , K. Kehrein , D. Riedel , M. Ott , S. Jakobs , Nat. Cell. Biol. 2018, 20, 528. 29662179 10.1038/s41556-018-0090-7

[57] H. Lemieux , S. Semsroth , H. Antretter , D. Hoefer , E. Gnaiger , Int. J. Biochem. Cell Biol. 2011, 43, 1729. 21871578 10.1016/j.biocel.2011.08.008

[58] M. Makrecka‐Kuka , G. Krumschnabel , E. Gnaiger , Biomolecules 2015, 5, 1319. 26131977 10.3390/biom5031319 PMC4598754

[59] K. P. Rentrop , F. Feit , Am. Heart J. 2015, 170, 971. 26542507 10.1016/j.ahj.2015.08.005

[60] G. D. Lopaschuk , J. R. Ussher , C. D. L. Folmes , J. S. Jaswal , W. C. Stanley , Physiol. Rev. 2010, 90, 207. 20086077 10.1152/physrev.00015.2009

[61] M. F. Berry , A. J. Engler , Y. J. Woo , T. J. Pirolli , L. T. Bish , V. Jayasankar , K. J. Morine , T. J. Gardner , D. E. Discher , H. L. Sweeney , Am. J. Physiol. Heart. Circ. Physiol. 2006, 290, H2196. 16473959 10.1152/ajpheart.01017.2005

[62] S. D. Prabhu , N. G. Frangogiannis , Circ. Res. 2016, 119, 91. 27340270 10.1161/CIRCRESAHA.116.303577 PMC4922528

[63] J. Rahman , S. Rahman , Lancet 2018, 391, 2560. 29903433 10.1016/S0140-6736(18)30727-X

[64] I. Martinez‐Reyes , N. S. Chandel , Nat. Commun. 2020, 11, 102. 31900386 10.1038/s41467-019-13668-3 PMC6941980

[65] R. Guo , J. Gu , S. Zong , M. Wu , M. Yang , Biomed J 2018, 41, 9. 29673555 10.1016/j.bj.2017.12.001 PMC6138618

[66] D. Nolfi‐Donegan , A. Braganza , S. Shiva , Redox. Biol. 2020, 37, 101674. 32811789 10.1016/j.redox.2020.101674 PMC7767752

[67] M. O. Isei , C. Kamunde , Biol. Med. 2020, 147, 114. 10.1016/j.freeradbiomed.2019.12.006 31825803

[68] M. Rubio‐Osornio , M. Orozco‐Ibarra , A. Diaz‐Ruiz , E. Brambila , M. C. Boll , A. Monroy‐Noyola , J. Guevara , S. Montes , C. Rios , Chem. Biol. Interact. 2017, 271, 1. 28442376 10.1016/j.cbi.2017.04.016

[69] S. Yoshikawa , A. Shimada , Chem. Rev. 2015, 115, 1936. 25603498 10.1021/cr500266a

[70] S. A. Moore , B. F. Anderson , C. R. Groom , M. Haridas , E. N. Baker , J. Mol. Biol. 1997, 274, 222. 9398529 10.1006/jmbi.1997.1386

[71] J. J. P. Stewart , J. Comput. Aided Mol. Des. 1990, 4, 1. 2197373 10.1007/BF00128336

[72] J. J. P. Stewart , J. Comput. Chem. 1991, 12, 320.

[73] A. Waterhouse , M. Bertoni , S. Bienert , G. Studer , G. Tauriello , R. Gumienny , F. T. Heer , T. A. P. de Beer , C. Rempfer , L. Bordoli , R. Lepore , T. Schwede , Nucleic Acids Res. 2018, 46, W296. 29788355 10.1093/nar/gky427 PMC6030848

[74] S. Pang , Y. Chen , C. Dai , T. Liu , W. Zhang , J. Wang , X. Cui , X. Guo , F. Jiang , Eur. J. Pharmacol. 2021, 907, 174303. 34217709 10.1016/j.ejphar.2021.174303

