# Supplementary material for: Copper‐loaded Milk‐Protein Derived Microgel Preserves Cardiac Metabolic Homeostasis After Myocardial Infarction
Source: Adv Sci (Weinh). 2024 Jul 15;11(35):2401527. doi: 10.1002/advs.202401527 (PMC11425262; doi:10.1002/advs.202401527)
Supplement: Supplementary file 1 — Supporting Information [file ADVS-11-2401527-s002.pdf]

Supplementary Materials for

Copper loaded milk-protein derived microgel preserves cardiac metabolic homeostasis after myocardial infarction

Xiaoqian Hong[1,2]†, Geer Tian[1,2,3]†, Binyao Dai[4]†, Xuhao Zhou[1,2], Ying Gao[1,2], Lianlian Zhu[1,2], Haoran Liu[5], Qinchao Zhu[6], Liwen Zhang[4], Yang Zhu[2,3,4], Daxi Ren[6], Chengchen Guo[5], Jinliang Nan[1,2], Xianbao Liu[1,2*], Jian'an Wang[1,2*], Tanchen Ren[1,2*]

[1]Department of Cardiology, the Second Affiliated Hospital, School of Medicine, Zhejiang University, Hangzhou, 310009, China.

[2]State Key Laboratory of Transvascular Implantation Devices, Heart Regeneration and Repair Key Laboratory Zhejiang Province, Hangzhou, 310009, China.

[3]Binjiang Institute of Zhejiang University, Hangzhou, 310053, China.

[4]MOE Key Laboratory of Macromolecular Synthesis and Functionalization, Department of Polymer Science and Engineering, Zhejiang University, Hangzhou, 310027, China.

[5]School of Engineering, Westlake University, Hangzhou, 310023, China.

[6]Institute of Dairy Science, College of Animal Sciences, Zhejiang University, Hangzhou, 310027, China.

†These authors contributed equally to this work.

*Corresponding authors.

Correspondence to

Tanchen Ren, Ph.D., Jian'An Wang, M.D., Ph.D., Xianbao Liu, Ph.D., Department of Cardiology of the Second Affiliated Hospital, State Key Laboratory of Transvascular Implantation Devices, Cardiovascular Key Laboratory Zhejiang Province, Zhejiang University School of Medicine, Hangzhou, 310009, China.

Email address: rentanchen120@zju.edu.cn (T. Ren), wangjianan111@zju.edu.cn (J. Wang), liuxb@zju.edu.cn (X. Liu)

This file includes:

Figures. S1 to S14

Table S1 and S2

Video S1

Experimental Section (Supplement)

Conductivity testing: Conductivity of casein hydrogel and Cu^{2+} loaded casein hydrogel were calculated from I-V curves (measured by 2400 SourceMeter, Keithley, with four-electrode method) by the formula:

$$\sigma = \frac{L}{R * S}$$

Here σ was electrical conductivity, L was electrode distance, R was resistivity, and S was the cross-sectional area of materials. Hydrogels with a size of 10 mm × 5 mm × 5 mm were used for this test.

CCK8 analysis and live/dead staining: Cells were seeded in the 96-well plates at a density of 40000 cells/ml, and the viability of cells treated with Cu^{2+} of different concentrations with or without OGD treatment was measured by CCK8 assays (Yeasen Biotechnology Co., Ltd., Shanghai, China) as previously reported. Calcein-AM/propidium iodide (PI) Live/Dead Cell Double staining Kit (Yeasen Biotechnology Co., Ltd., Shanghai, China) was used to test the cytotoxicity of Cu^{2+} at different concentrations with or without OGD injury.

Tube formation assays and VEGF concentration detection: For tube formation assay, 96-well plates were coated with 40 μl Matrigel (Corning, USA) per well and incubated at 37 °C for 1 h. GFP-HUVECs purchased from iCell (Shanghai, China) were seeded at a density of 10000 cells/well in high-glucose DMEM without FBS containing Cu^{2+} or CuCMGs with different concentrations, and incubated for 3-4 h in a 37 °C hypoxia incubator with 0.3% O_2. Capillary-like structure formation by GFP-HUVECs was recorded with fluorescent microscope. Image J with a plugin named Angiogenesis Analyzer was used to quantify the tube length. The supernatant of each group

was collected and VEGF concentration was detected using a human VEGF ELISA kit (MultiSciences, Hangzhou, China).

Histotoxicity analysis: To visualize and and analyze the histotoxicity in the MI rats after the treatment of microgels or Cu^{2+} at Day 28 after injection, the slices livers, spleens, kidneys and lungs were stained with hematoxylin aqueous solution and aqueous eosin Y solution (H&E staining, Sigma-Aldrich, Germany).

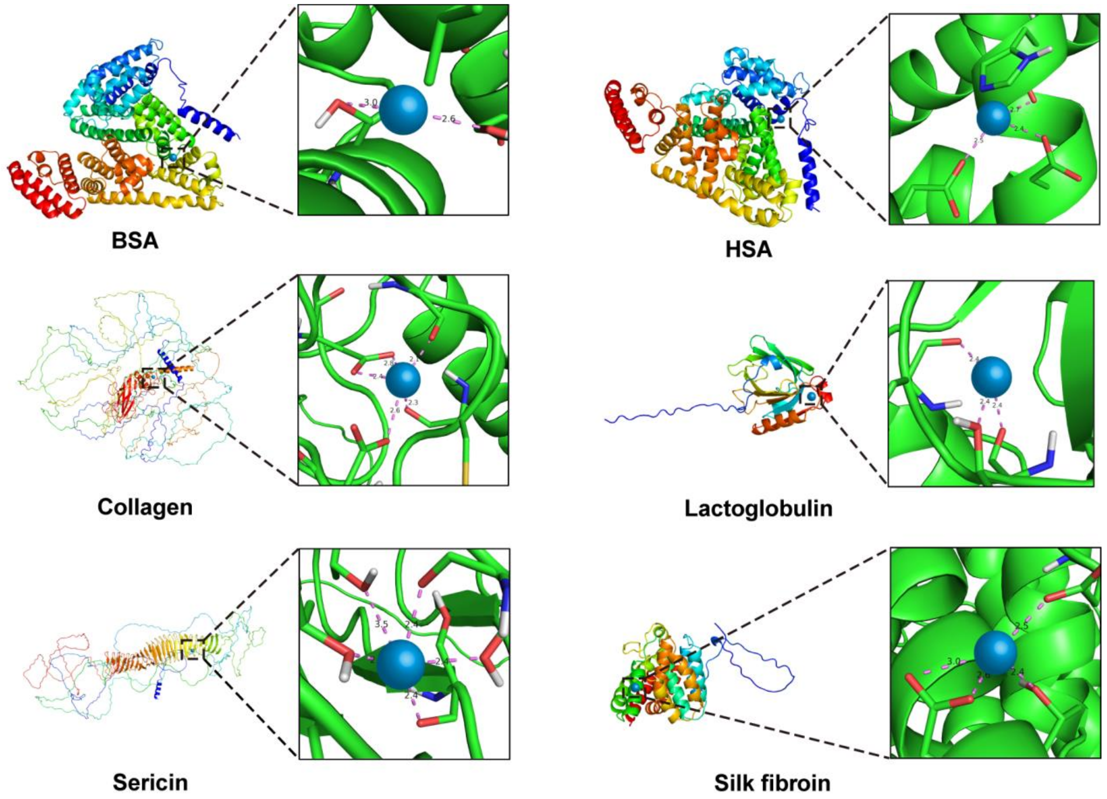

Figure S1. Schematic diagram of molecular docking between other proteins and Cu^{2+}.

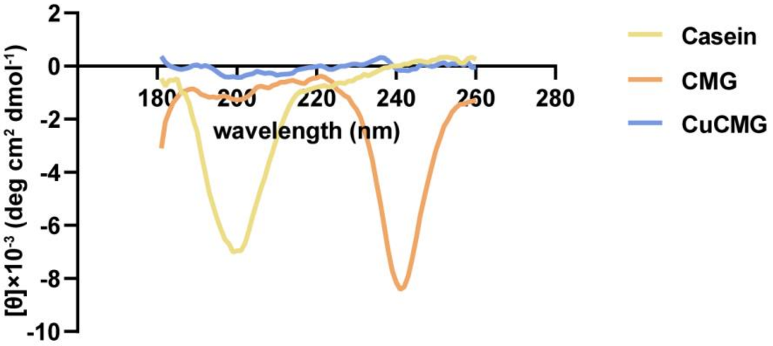

Figure S2. Circular dichroism spectrum of Casein, CMG, and CuCMG.

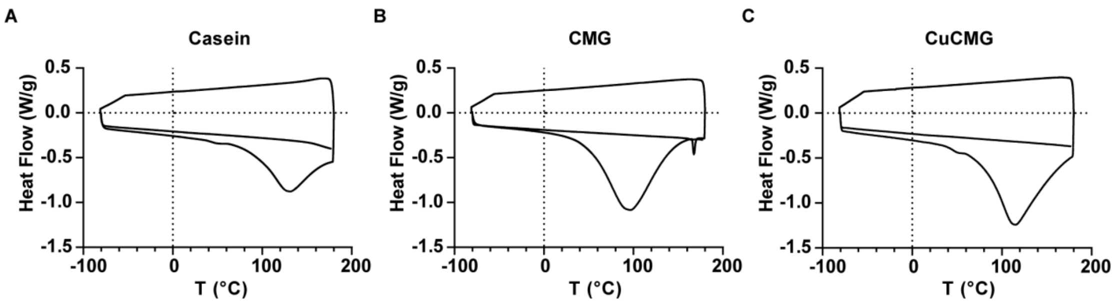

Figure S3. Differential scanning calorimetry analysis results of Casein (A), CMG (B) and CuCMG (C).

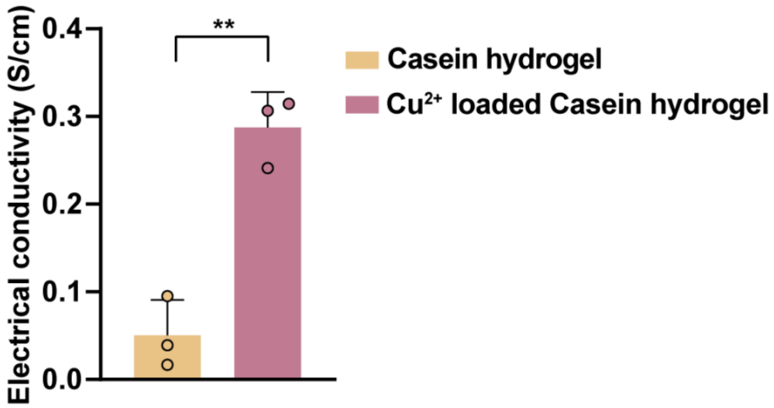

Figure S4. Electrical conductivity of casein hydrogel and Cu^{2+} loaded casein hydrogel. **P < 0.01.

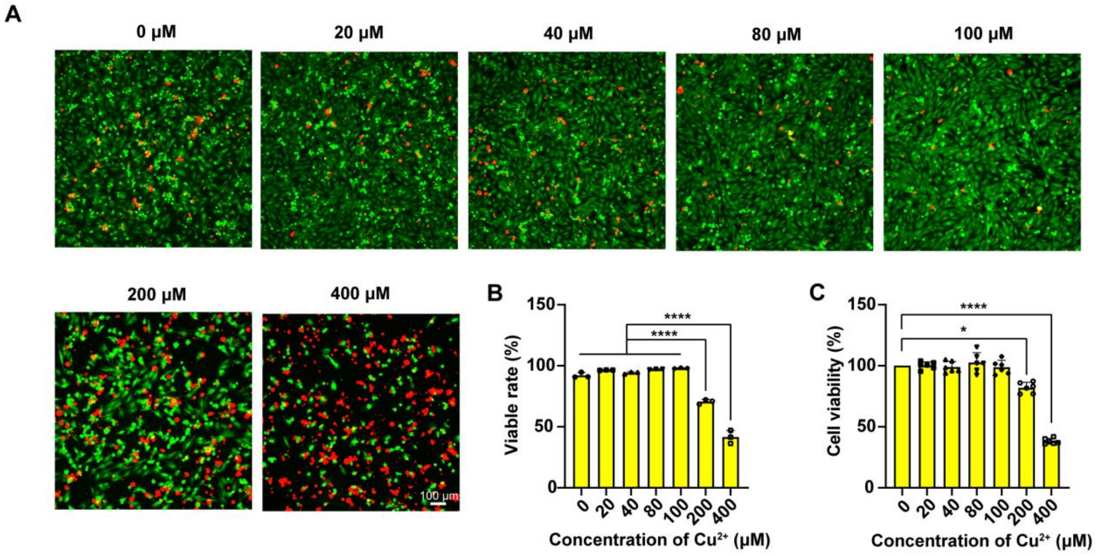

Figure S5. The effect of different concentrations of Cu^{2+} on the activity of H9C2 cells under normoxic conditions. A) H9C2 cells in the medium supplemented with Cu^{2+} at different concentrations under normoxic conditions analyzed by live/dead staining. Live cells were green, and dead cells were red. B) Statistics of the viable rate of the H9C2 cells. C) The cell viability of

H9C2 cells measured by CCK8 after culturing in the medium supplemented with Cu^{2+} at different concentrations normalized to no Cu treated. *P < 0.05, **P < 0.01, ***P < 0.001 and ****P < 0.0001.

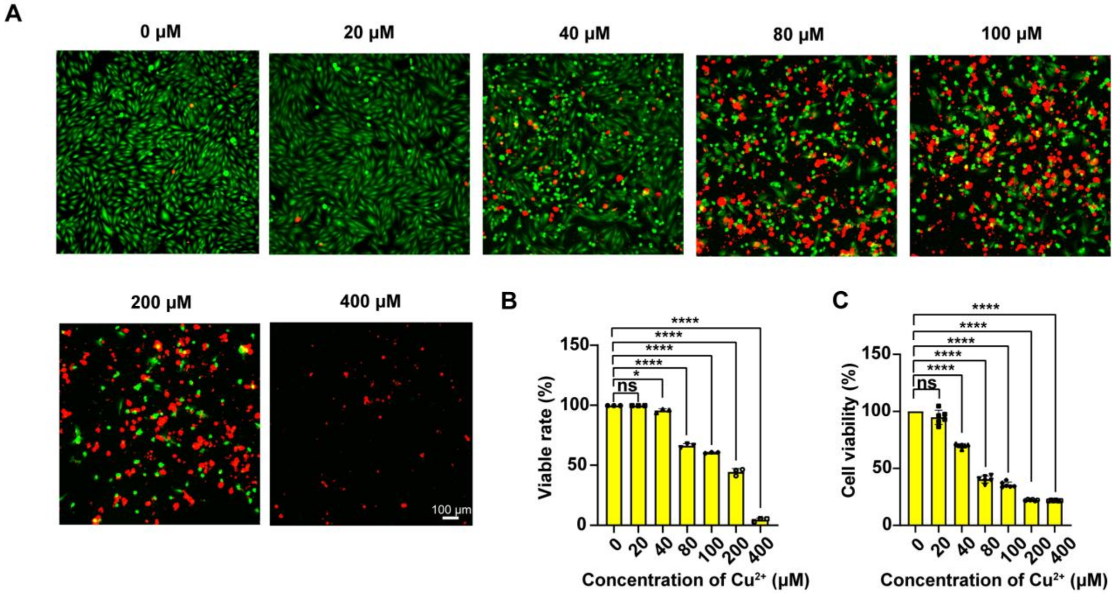

Figure S6. The effect of different concentrations of Cu^{2+} on the activity of H9C2 cells under hypoxic conditions. A) H9C2 cells in the medium supplemented with Cu^{2+} at different concentrations after OGD injury analyzed by live/dead staining. Live cells were green, and dead cells were red. B) Statistics of the viable rate of the H9C2 cells. C) The cell viability of H9C2 cells measured by CCK8 after culturing in the medium supplemented with Cu^{2+} at different concentrations after OGD injury. *P < 0.05, **P < 0.01, ***P < 0.001 and ****P < 0.0001.

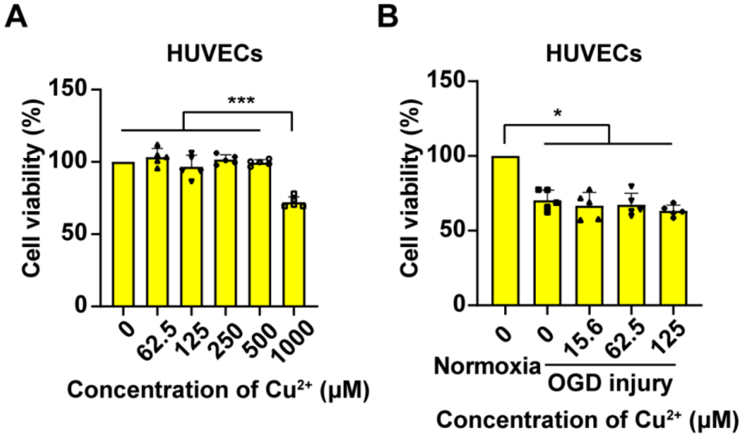

Figure S7. The viability of HUVECs measured by CCK8 in the medium supplemented with different concentrations of Cu^{2+} with (B) or without (A) OGD injury.

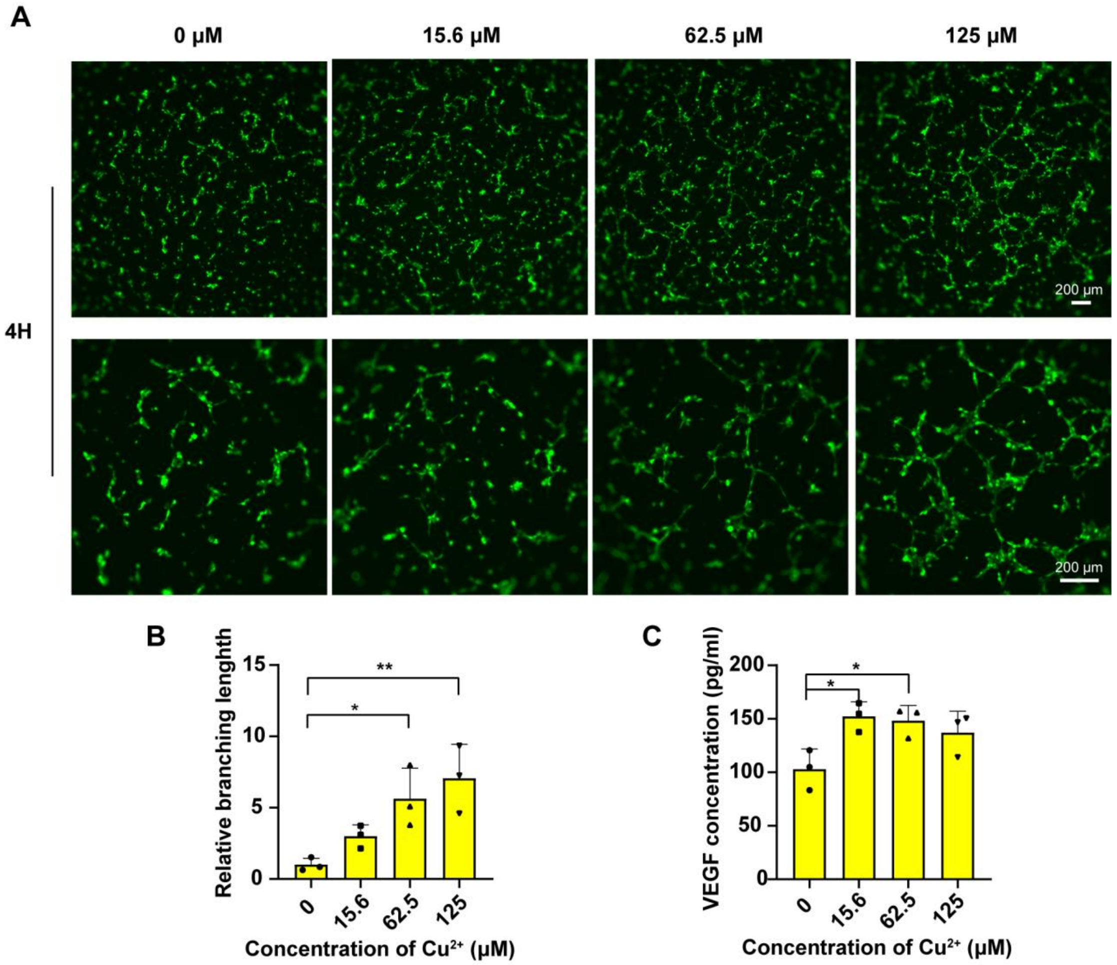

Figure S8. Cu^{2+} stimulate angiogenic effects of HUVECs. A) Images and B) quantitative analysis of HUVEC tube formations under different concentrations of Cu^{2+} for 4 hours. Scale bars = 200 μm. C) VEGF secretion of HUVECs cultured under different concentrations of Cu^{2+} for 4 hours. *P < 0.05, **P < 0.01.

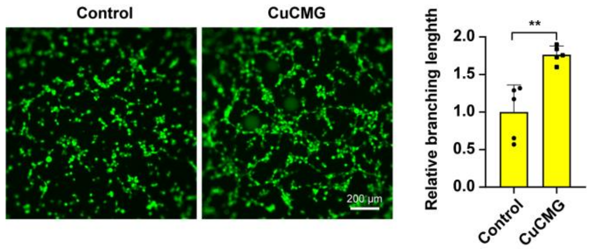

Figure S9. Images and quantitative analysis of HUVEC tube formations with or without CuCMGs. The concentration of Cu^{2+} released by CuCMGs within 3-4 h was ~15 μM. Scale bars = 200 μm. **P < 0.01.

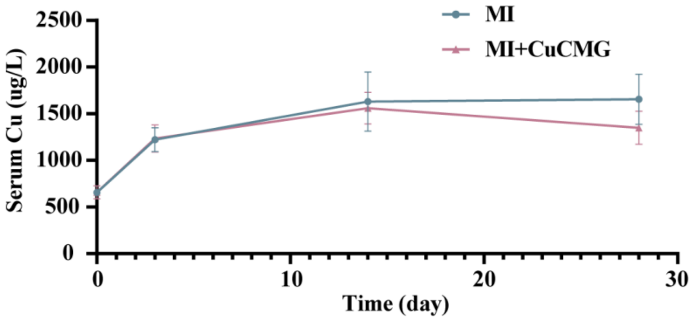

Figure S10. Serum Cu^{2+} levels at different time points after MI. n=3.

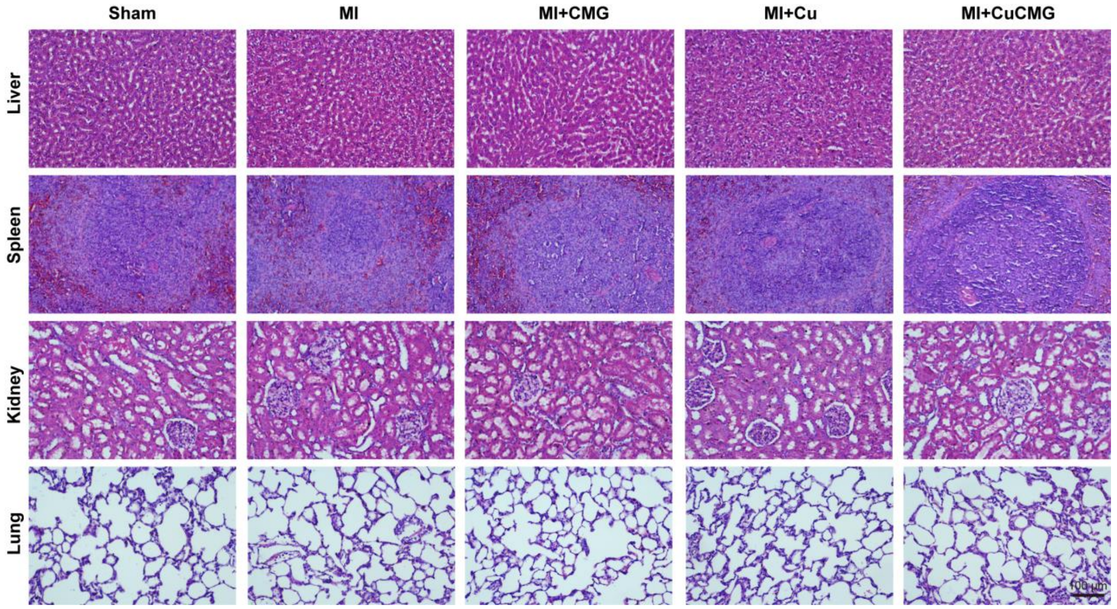

Figure S11. H&E staining images of the main organs resected from different groups at Day 28. Scale bars = 100 μm.

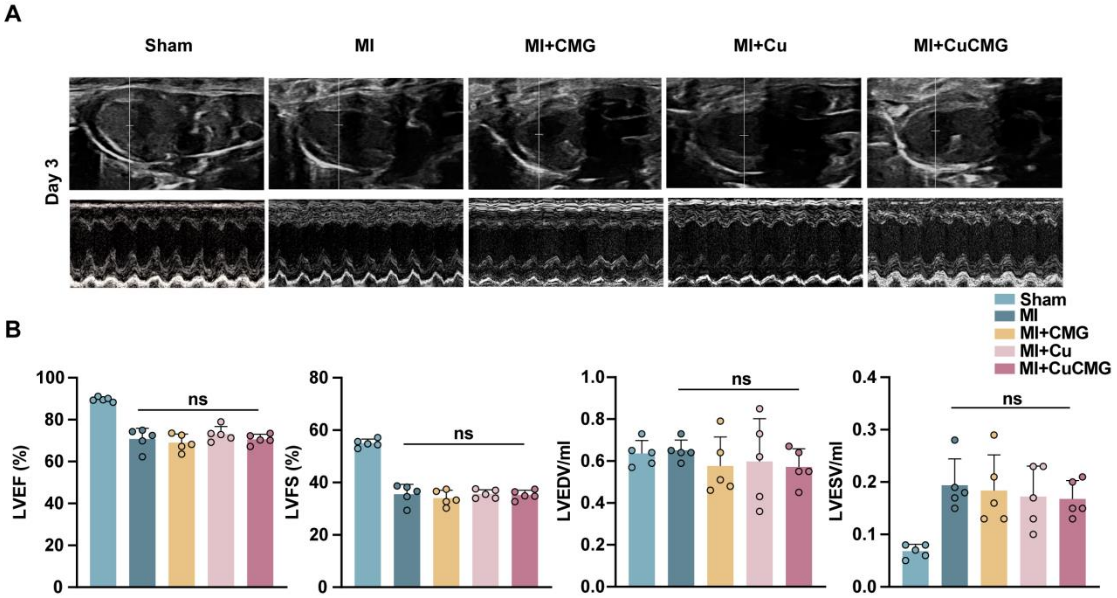

Figure S12. Cardiac function at Day 3. A) Representative echocardiograms obtained from the mid-papillary muscle region of the left ventricle of rats at Day 3 after MI. B) Echocardiography analysis of LVEF, LVFS, LVEDV, and LVESV at Day 3 after injection.

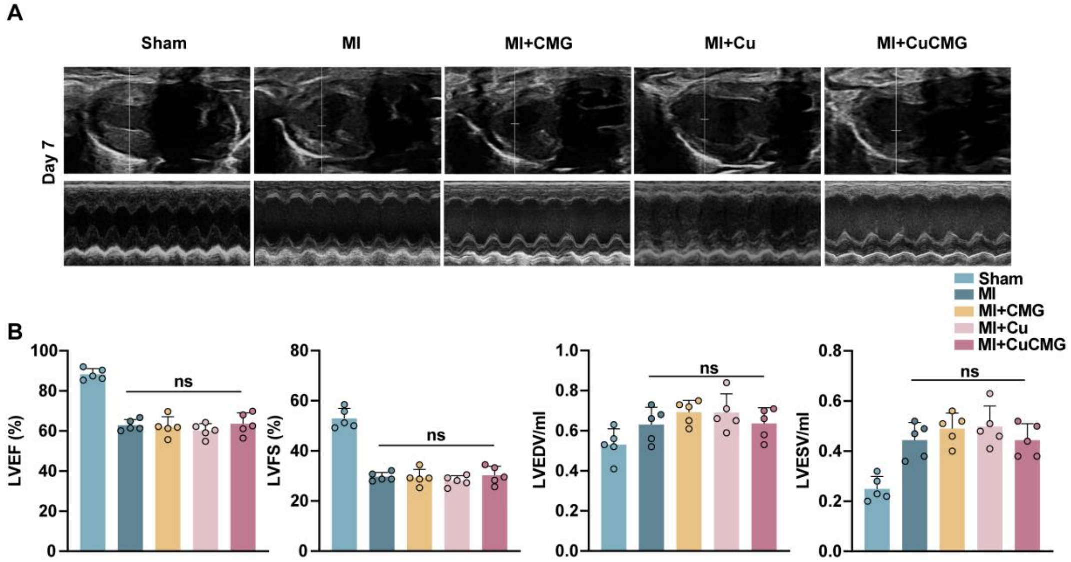

Figure S13. Cardiac function at Day 7. A) Representative echocardiograms obtained from the mid-papillary muscle region of the left ventricle of rats at Day 7 after MI. B) Echocardiography analysis of left ventricular ejection fraction (LVEF), left ventricular fractional shortening (LVFS), left ventricular end-diastolic volume (LVEDV), and left ventricular end-systolic volume (LVESV) at Day 7 after injection.

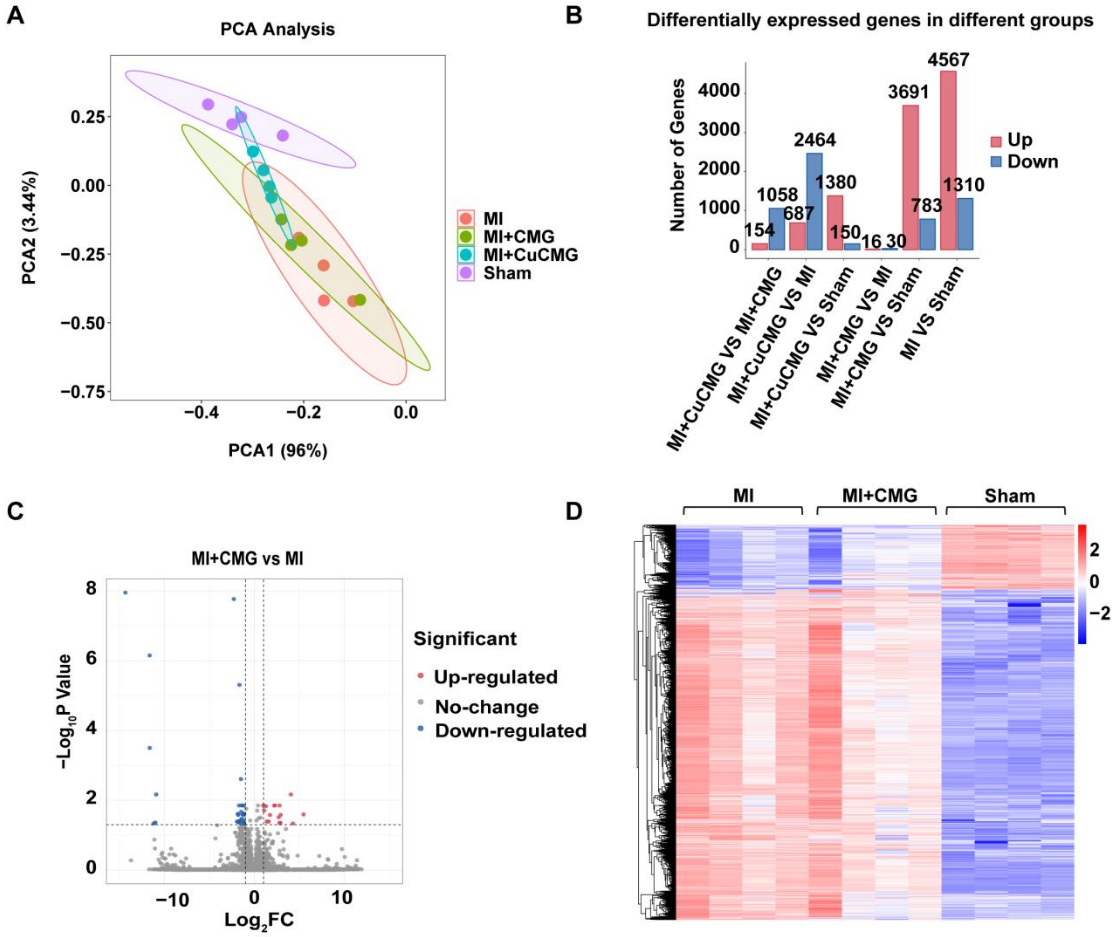

Figure S14. RNA-seq analysis of the tissue samples at Day 3 post-surgery in MI vs. MI+CMG vs. MI+CuCMG vs. Sham hearts. A) Principal component analysis (PCA) of transcriptomes of the infarct area from the MI, MI+CMG, MI+CuCMG and Sham groups. B) Differentially expressed genes in different groups. C) Volcano plot of all of the expressed genes from the MI and MI+CMG group. (|Log2Fold Change|>1, adjusted P Value<0.05). D) Hierarchical clustering of differentially expressed genes in MI vs. MI+CMG vs. Sham hearts as assessed by RNA-seq. n=4 rats per group.

Table S1

Primer sequences for the genes observed in this study

Gene	Forward primer	Reverse primer
HIF1A	TATGAGCCAGAAGAACTTTTAGGC	CACCTCTTTTGGCAAGCATCCTG
VEGFA	TTGCCTTGCTGCTCTACCTCCA	GATGGCAGTAGCTGCGCTGATA
FGF2	AGCGGCTGTACTGCAAAAACGG	CCTTTGATAGACACAACTCCTCTC
ACTB	CACCATTGGCAATGAGCGGTTC	AGGTCTTTGCGGATGTCCACGT
Postn	AACAACTCCGTGTCTTCGTGTATCG	CCTGCTTGCTTCCTCTCACCATG
Col1a1	TGTTGGTCCTGCTGGCAAGAATG	GTCACCTTGTTCGCCTGTCTCAC
Ccn2	GCCAGGGAGTAAGGGACACG	CCCTCCCCTGTCACACTCCAAA
Cs	GAACTCATCCTGCCTCGTCCTTG	CTGTCTTCCCATGCTGCTGTCTG
Idh2	GTGGCTCAGGTGCTCAAGTCTTC	GCTTCAATGGTCTTCCCGTCAGG
Ndufs2	CTCCGAGGCTCAGGCATCCAG	CGCATCTCTTCCACACGACACAG
Sdha	GCTCTTTCCTACCCGCTCACATAC	GCCATCTCCAGTTGTCCTCTTCC
Uqcrfs1	ACCAAGAAGGAGATTGACCAGGAAG	ACCAAGATGAGTACAGACACCGATC

Cox4a	CCCACGTCAAGCTGCT GTCTG	CCCACCACTGTCTTCC ACTCATTG
Atp5a1	CTGTTGCTTACCGCCA GATGTCTC	AAAGAGCCACCACCA AAGGAATCG
Gapdh	ACGGCAAGTTCAACG GCACAG	CGACATACTCAGCAC CAGCATCAC

Table S2

Primary antibodies used for WB in this study

Antigen	Company	Catalog #	Origin	Working dilution
Cs	Peoteintech	67784-Hg	Mouse	1:2000 for WB
Idh2	HUABIO	ET1704-93	Rabbit	1:2000 for WB
Ndufs2	HUABIO	HA721968	Rabbit	1:2000 for WB
Sdha	HUABIO	ET1703-40	Rabbit	1:2000 for WB
Uqcrfs1	HUABIO	ET7108-28	Rabbit	1:2000 for WB
Cox4a	Peoteintech	11242-1-Ap	Rabbit	1:10000 for WB
Atp5a1	HUABIO	ET1703-53	Rabbit	1:2000 for WB

Video S1. Morphological changes of CMGs during the adsorption of Cu^{2+}. 2mM Cu^{2+} solution was dropped from the upper right corner of the screen. Scale bars = 50 μm (Vedio is demonstrated at speed 18×).
